# *MYC* Cooperates with *AKT* in Prostate Tumorigenesis and Alters Sensitivity to mTOR Inhibitors

**DOI:** 10.1371/journal.pone.0017449

**Published:** 2011-03-04

**Authors:** Nicola J. Clegg, Suzana S. Couto, John Wongvipat, Haley Hieronymus, Brett S. Carver, Barry S. Taylor, Katharine Ellwood-Yen, William L. Gerald, Chris Sander, Charles L. Sawyers

**Affiliations:** 1 Human Oncology and Pathogenesis Program, Memorial Sloan-Kettering Cancer Center, New York, New York, United States of America; 2 Laboratory of Comparative Pathology, Memorial Sloan-Kettering Cancer Center, New York, New York, United States of America; 3 Howard Hughes Medical Institute, Memorial Sloan-Kettering Cancer Center, New York, New York, United States of America; 4 Department of Surgery and Urology Service, Memorial Sloan-Kettering Cancer Center, New York, New York, United States of America; 5 Computational Biology Center, Memorial Sloan-Kettering Cancer Center, New York, New York, United States of America; 6 Department of Molecular and Medical Pharmacology, David Geffen School of Medicine, University of California Los Angeles, Los Angeles, California, United States of America; Sanford-Burnham Medical Research Institute, United States of America

## Abstract

*MYC* and phosphoinositide 3-kinase (PI3K)-pathway deregulation are common in human prostate cancer. Through examination of 194 human prostate tumors, we observed statistically significant co-occurrence of *MYC* amplification and PI3K-pathway alteration, raising the possibility that these two lesions cooperate in prostate cancer progression. To investigate this, we generated bigenic mice in which both activated human AKT1 and human MYC are expressed in the prostate (MPAKT/Hi-MYC model). In contrast to mice expressing AKT1 alone (MPAKT model) or MYC alone (Hi-MYC model), the bigenic phenotype demonstrates accelerated progression of mouse prostate intraepithelial neoplasia (mPIN) to microinvasive disease with disruption of basement membrane, significant stromal remodeling and infiltration of macrophages, B- and T-lymphocytes, similar to inflammation observed in human prostate tumors. In contrast to the reversibility of mPIN lesions in young MPAKT mice after treatment with mTOR inhibitors, Hi-MYC and bigenic MPAKT/Hi-MYC mice were resistant. Additionally, older MPAKT mice showed reduced sensitivity to mTOR inhibition, suggesting that additional genetic events may dampen mTOR dependence. Since increased MYC expression is an early feature of many human prostate cancers, these data have implications for treatment of human prostate cancers with PI3K-pathway alterations using mTOR inhibitors.

## Introduction

Prostate cancer is the second most common cause of cancer-related deaths in American men, who carry a 16% lifetime risk of developing invasive prostate cancer. Effective treatment of early-stage localized disease involves active surveillance, surgery (radical prostatectomy) or radiation therapy; however, recurrent and/or metastatic disease is incurable and androgen deprivation therapy is the primary treatment modality [1], [2]. The predominant genetic and cellular changes in human prostate cancer include presence of the *TMPRSS2-ERG* gene fusion [3]; loss of the phosphatase and tensin homolog (*PTEN*) tumor suppressor gene leading to accumulation of its substrate phosphatidylinositol 3,4,5-triphosphate (PIP_3_) and constitutive PI3K-pathway up-regulation [4]; amplification, over-expression or mutation of the androgen receptor (AR) [2]; and amplification of the *MYC* oncogene [5], [6]. Activating mutations in some signaling pathways can lead to tumor cell ‘addiction’ to that same pathway, providing an Achilles heel for clinical intervention.

The PI3K-pathway activates multiple targets including AKT and its downstream effector mammalian target of rapamycin (mTOR) [7], [8], thus promoting cell growth and survival by suppression of apoptosis and modulation of glucose uptake and cellular metabolism [9]. mTOR function is governed by its participation in the mTORC1 (mTOR complex 1) and mTORC2 (mTOR complex 2) multiprotein complexes [7], [8]. AKT is one of several mTORC2 kinase substrates, whereas activated mTORC1 phosphorylates two key effectors: i) eukaryotic initiation factor 4E–binding protein 1 (4EBP1) that regulates cap-dependent protein translation; and ii) ribosomal protein S6 kinase 1 (S6K1) that in turn phosphorylates 40S ribosomal protein S6, leading to protein synthesis.

PI3K-pathway inhibitors are undergoing clinical evaluation in multiple tumor types [10] including prostate cancer [11]. Despite promising preclinical efficacy in PI3K-pathway-dependent prostate cancer models [12], [13], [14], there have been only sporadic clinical responses in single-agent trials with rapamycin analogs (rapalogs, eg CCI-779, RAD001) targeting the PI3K-pathway *via* allosteric inhibition of mTORC1 [15], [16]. One reason for the limited clinical efficacy of mTOR inhibitors could be a compensatory upregulation of PI3K signaling to mitigate the inhibitory block placed on the rapamycin-sensitive mTORC1 complex, either *via* release of the negative feedback on AKT that is potentiated by activated S6K in the absence of rapamycin, or *via* mTORC2 signaling, which is largely insensitive to rapamycin [17], [18], [19]. Additionally, mTORC1 inhibition can lead to feedback activation of mitogen-activated protein kinase (MAPK) signaling *via* an S6K-PI3K-Ras-dependent pathway [20]. Furthermore, rapamycin does not fully inhibit mTORC1, as demonstrated by comparison with ATP-competitive mTOR kinase inhibitors [16]. Another explanation for rapalog failure in the clinic is that tumorigenesis depends on accumulation of more than one genetic aberration in pathways regulating cell proliferation and survival [21]. Elucidation of these cooperating lesions is essential to development of effective therapeutic strategies.

The MYC transcription factor directly regulates expression of the translational machinery for protein synthesis, as well as genes controlling cell cycle progression, metabolism, mitochondrial number and function and stem cell self renewal [22]. A potential cooperative role for PI3K-pathway activation and the *MYC* oncogene has not yet been documented in human prostate cancer, although pathway-interaction has been suggested by several *in vitro* and *in vivo* models [23], [24], [25].

We identified an association between PI3K-pathway alteration and *MYC* amplification in a cohort of primary and metastatic human prostate cancer samples. To explore a cooperative role for the PI3K-pathway with the *MYC* oncogene in human prostate cancer, we used existing murine models of human prostate cancer harboring prostate-specific homozygous deletion of *PTEN* (PTEN^pc−/−^ model) [26], [27], or over-expression of either human MYC (Hi-MYC model) [28] or the downstream PI3K-pathway active allele of *AKT1* (MPAKT model) [29] and studied the combinatorial effect of these pathways on tumorigenesis. Initial generation of a PTEN^pc−/−^/Hi-MYC bigenic cross was used to validate results of a related study [24] that demonstrated an interaction between *PTEN* and *MYC* signaling using prostate-specific deletion of *PTEN* with concurrent Cre-induced focal MYC expression to induce high-grade mPIN (HG-mPIN) lesions and invasive adenocarcinoma. To address whether *AKT* downstream of *PTEN* might be the key mediator, we further explored the cooperation between these pathways using a bigenic mouse cross, MPAKT/Hi-MYC. Treatment with an mTOR inhibitor allowed direct assessment of the impact of MYC expression on the well-documented sensitivity of prostate lesions in the activated AKT model [14]. Our results suggest the disappointing clinical activity of single-agent rapamycin analogs in *PTEN*-deficient human cancers, as compared to single-lesion transgenic mouse models, may arise from secondary genetic alterations in human tumors.

## Materials and Methods

Detailed methods are provided as supplemental information (Text S1).

### Ethics statement

Human prostate tissues analyzed in this study were from patients treated at Memorial Sloan-Kettering Cancer Center (MSKCC), all of whom provided written informed consent. The study was approved by the MSKCC Institutional Review Board and the MSKCC Human Tissue Utilization Committee. Animal studies were carried out under protocol 06-07-012 approved by the MSKCC Institutional Animal Care and Use Committee. Institutional guidelines for the proper, humane use of animals in research were followed.

### Comparative Genomic Hybridization Analysis of human tumors

Copy number data from 194 high-quality primary and metastatic tumors were generated using the Agilent 244K aCGH array, and tumors assessed for genomic gain or amplification in *MYC*, *PIK3CA*, *AKT1*, *AKT2* and *AKT3*, and for *PTEN-*loss (Table S1). The complete aCGH dataset is reported separately [30] and available online at http://cbio.mskcc.org/prostate-portal/.

### Generation, treatment and characterization of PTEN^pc−/−^/Hi-MYC and MPAKT/Hi-MYC mice

PTEN^pc−/−^ mice (Pten^loxP/loxP^/Pb-Cre4) have been described [26], [27]. Hi-MYC mice (ARR2PB-Flag-*MYC*-PAI transgene) [28] were crossed with PTEN^loxP/loxP^ mice [26], [27], and PTEN^loxP/loxP^/Hi-Myc offspring (F2) crossed with PTEN^loxP/wt^/Pb-Cre4 males [26], [27], [31] generating bigenic PTEN^pc−/−^/Hi-MYC mice (F3). MPAKT (rPb-myr-HA-*AKT1* transgene) [29] and Hi-MYC mice were cross-bred to generate MPAKT/Hi-MYC mice. Males in treatment cohorts were dosed qd with either 10 mg/kg (po) RAD001 emulsion or placebo (Novartis Pharma AG) for 14d, unless otherwise noted. Tissues were stained for histologic or immunohistochemical analysis; imaged slides are available online at http://cbio.mskcc.org/Public/Sawyers_Clegg_AktMyc_2010. Gene and protein expression were assessed by quantitative real-time RT-PCR and immunoblot.

## Results

### MYC amplification co-occurs with PI3K-pathway activation in human prostate tumors

Activation of the PI3K signaling pathway, often via *PTEN* inactivation, and amplification of *MYC* are common genetic alterations in prostate cancer that correlate with high histological grade and poor prognosis [5], [32]. To evaluate whether PI3K-pathway activation and *MYC* oncogene amplification co-occur in human prostate cancer, we examined oligonucleotide array CGH data from 194 prostate tumors, including 37 metastases. PI3K-pathway activation rarely occurred through point mutation of *PTEN* or *PIK3CA* in this dataset: exon-resequencing of 80 tumors revealed only 2 tumors with *PIK3CA* mutation and none with *PTEN* mutation [30].

**Figure 1:**
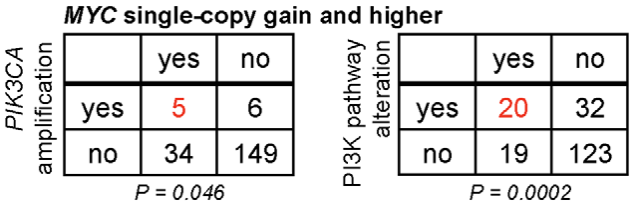
*MYC* amplification co-occurs with AKT pathway activation in human prostate tumor samples. Contingency tables for co-occurrence of *MYC* and PI3K-pathway copy-number-alterations (defined by aCGH) from 157 primary and 37 metastatic prostate tumor samples. Indicated in *red* is the proportion of tumors (n = 194 total) with *PIK3CA* amplification (*left*) or general PI3K-pathway gain (*right*) that also exhibit single- or multi-copy *MYC* gain. Statistical significance for each association is reported as a P-value (2-tailed Fisher's exact test).

PI3K-pathway activation, representing combinatorial alterations in *PTEN*, *PIK3CA*, *AKT1*, *AKT2* and *AKT3* (single- or multi-copy), was found in 27% of all samples and 70% of metastases. *MYC* multi-copy gain was identified in 6% of all samples and 24% of metastases, increasing to 20% of all samples and 51% of metastases when both single- and/or multi-copy *MYC* gain are considered (Table S1) [5], [6], [32]. We examined whether tumors harboring PI3K-pathway alteration were enriched for *MYC* copy-number gain (single-copy or greater) and found a positive association (P = 0.0002, Fisher's two-tailed exact test; Figs. 1, S2A). Enrichment of PI3K-pathway copy-alterations and high-level *MYC* amplification was also observed (P = 0.0004; Figs. S1A, S2A). The subset of tumors with specific *PIK3CA* amplification also showed an association with *MYC* amplification (Figs. 1, S1A, S2A). There was also a statistically significant association between PI3K-pathway alterations and *MYC* multi-copy gain in metastases (P = 0.036, Figs. S1B–C, S2B). These data establish that alterations in the PI3K-pathway are enriched with *MYC* amplification in human prostate tumors.

### MYC and AKT cooperate to accelerate progression of mPIN to invasion in a murine prostate cancer model

To assess the functional implications of the association between PI3K-pathway alteration and *MYC* amplification in human prostate tumors, we turned to genetically engineered mouse (GEM) models. The role of PI3K signaling in prostate cancer has been modeled in mice by deletion of *PTEN* or by transgenic expression of activated AKT, while the role of *MYC* has been investigated by transgenic expression of MYC. A recent study demonstrated interaction between *PTEN* and *MYC* signaling using prostate-specific hetero- or homozygous deletion of *PTEN* with concurrent focal probasin-Cre-driven MYC overexpression [24]. In order to validate this result in a model with widespread prostate-specific MYC expression, and provide rationale for more extensive studies of the role of AKT, we employed the Hi-MYC [28] transgenic model (FVB background) in a bigenic cross with the prostate-specific PTEN^pc−/−^ conditional knockout mouse (C57BL/6J strain) [26], [27] to generate bigenic PTEN^pc−/−^/Hi-MYC mice.

In the Hi-MYC model [28], the modified (ARR_2_PB) probasin promoter-driven expression of human MYC in the prostate results in murine prostate intraepithelial neoplasia (mPIN) in the lateral prostate (LP) by 4 weeks of age that progresses to adenocarcinoma in all mice by 6–9 months. The ventral prostate (VP), dorsal prostate (DP) and anterior prostate (AP) are affected to a lesser extent. The PTEN^pc−/−^ model expresses probasin-Cre4 (Pb-Cre4) [31] upon puberty, thereby inactivating the floxed *PTEN* alleles in the VP, LP, DP and AP. PTEN^pc−/−^ mice develop HG-mPIN that progresses to invasive adenocarcinoma after ∼6 months of age [26], [27].

**Figure 2:**
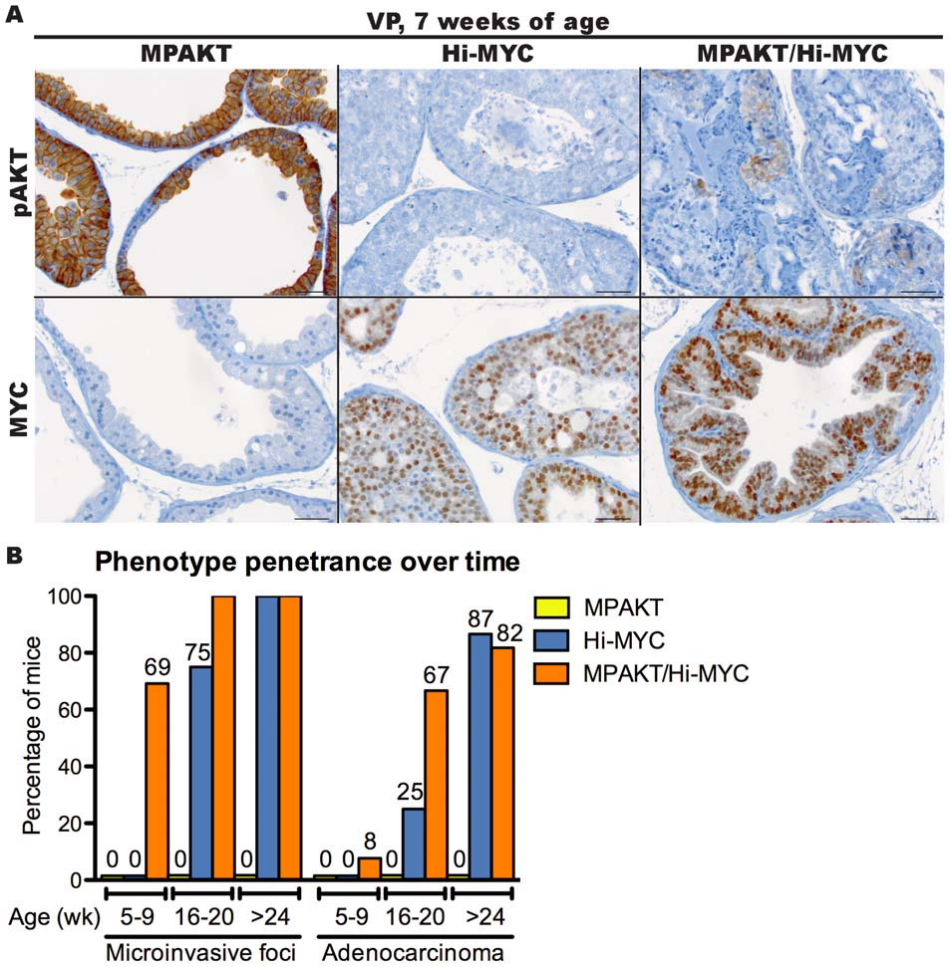
*MYC* cooperates with PI3K-pathway signaling in bigenic PTEN^pc−/−^/Hi-MYC mice resulting in invasive adenocarcinoma after 3 months. Mouse prostates (3–4 months), showing high-grade mPIN within glands of the PTEN^pc−/−^ and Hi-MYC prostates, and adenocarcinoma in the PTEN^pc−/−^/Hi-MYC prostate. *Upper panels*: hematoxylin-and-eosin (H&E). *Lower panels*: Immunohistochemistry (IHC). Note cellular expression of both pAKT and MYC in invasive areas of the PTEN^pc−/−^/Hi-MYC prostate (*dashed line*), and basement membrane disruption illustrated by loss of collagen IV staining. Scale-bars: 200 µm (black), 400 µM (red).

PTEN^pc−/−^/Hi-MYC bigenic mice have large prostatic adenocarcinomas at 3 months (Fig. 2), well in advance of either of the well-established single lesion models, which at this stage harbor mPIN exclusively (Fig. 2). Assessment of expression patterns for pAKT (increased by inactivation of *PTEN*) and MYC in the PTEN^pc−/−^/Hi-MYC prostatic epithelium revealed a subpopulation of cells expressing both proteins at high levels in areas of invasion (Fig. 2). Consistent with previous work [24], PI3K-pathway activation and *MYC* cooperate to accelerate progression of invasive prostate cancer, providing the rationale to characterize this cooperation more extensively and in a pure genetic background.

To address whether *AKT*, downstream of *PTEN*, might be responsible for the interaction between PI3K-pathway activation and *MYC* signaling, and whether mTOR is a key mediator, we selected the established MPAKT [29] and Hi-MYC [28] transgenic models, both in the FVB background strain, and cross-bred them to generate MPAKT/Hi-MYC mice with prostate-specific expression of both transgenes. In the MPAKT model, over-expression of myristoylated human AKT1, driven by a portion of the prostate-specific rat probasin promoter, leads to phospho-AKT expression in luminal epithelial cells of predominantly the VP and rarely the LP. Expression of activated AKT correlates with a highly-penetrant phenotype of mPIN in mice by 6–8-weeks-old [29].

**Figure 3:**
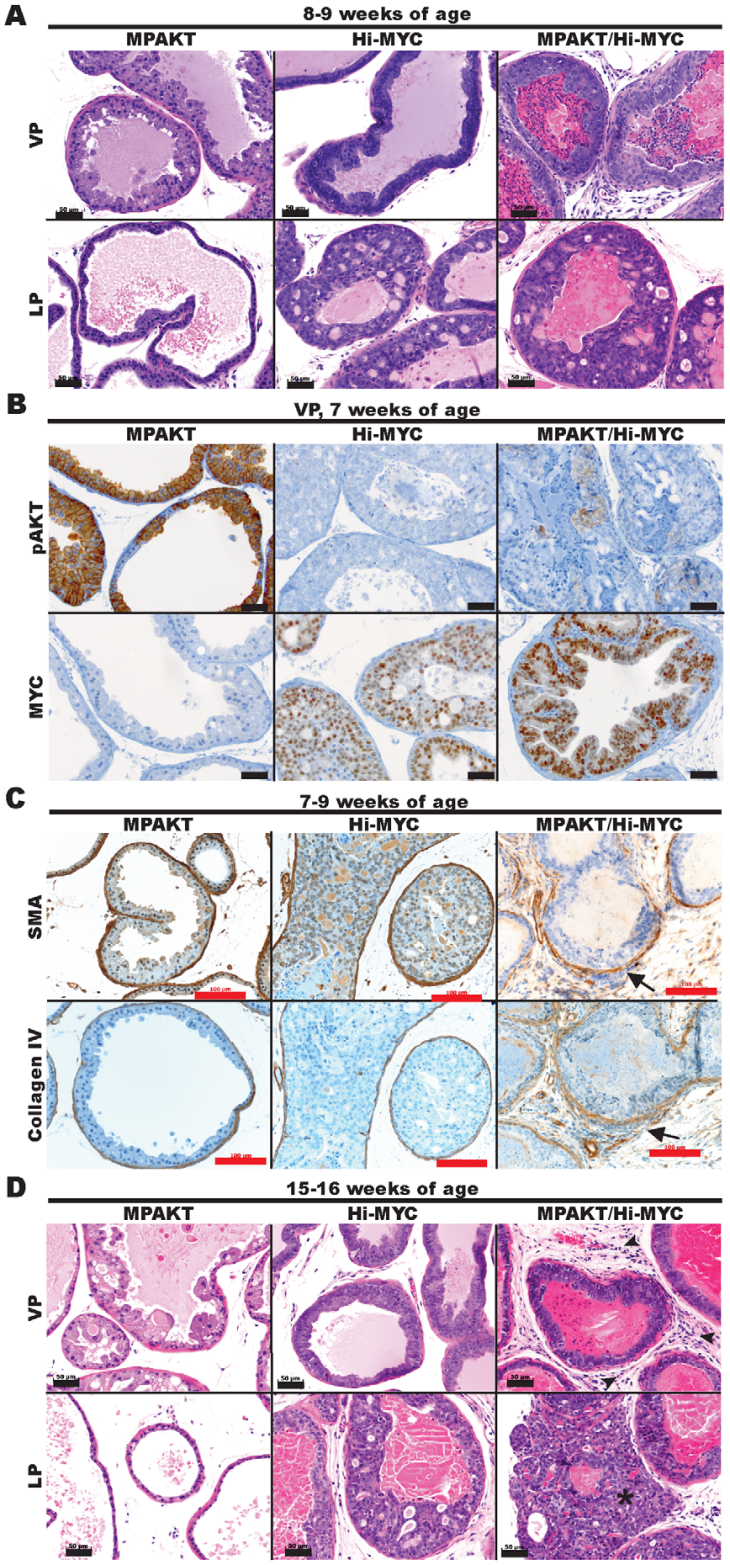
*MYC* cooperates with *AKT* in the MPAKT/Hi-MYC model resulting in extensive stromal remodeling typical of microinvasion by 5–9 weeks. Mouse prostates at 7–9-weeks-old (A–C) or 15–16-weeks-old (D). (A) MPAKT has normal glands in lateral prostate (LP, *lower panels*) and large areas of mPIN in ventral prostate (VP, *upper panels*); Hi-MYC and MPAKT/Hi-MYC have diffuse mPIN in VP (*upper panels*) and LP (*lower panels*) (H&E). Scale-bars: 50 µm. (B) IHC indicating that *AKT* and *MYC* transgenes are expressed in prostates of bigenic MPAKT/Hi-MYC mice, albeit at lower levels than in single lesion mice. High levels of pAKT membrane staining are associated with regions of mPIN in VP of MPAKT and are slightly less intense and patchier in MPAKT/Hi-MYC, but absent in Hi-MYC. Nuclear MYC staining is evident in Hi-MYC and MPAKT/Hi-MYC VP, but absent in MPAKT mice. Scale-bars: 50 µm. (C) IHC for SMA and collagen IV. Note disrupted and absent smooth muscle stroma and collagen IV in MPAKT/Hi-MYC (*right panels*), compared to minimally attenuated smooth muscle sheath and collagen IV around areas of mPIN in Hi-MYC (*center panels*); smooth muscle sheath remains intact in mPIN from MPAKT (*left panels*). Scale-bars: 100 µm. (D) Normal tissue (LP) and mPIN (VP) in MPAKT; diffuse mPIN in VP and LP of Hi-MYC and MPAKT/Hi-MYC; frank adenocarcinoma (*asterisk*) in LP of MPAKT/Hi-MYC; stromal inflammation in MPAKT/Hi-Myc (*arrowheads*) (H&E). Scale-bars: 50 µm.

**Figure 4:**
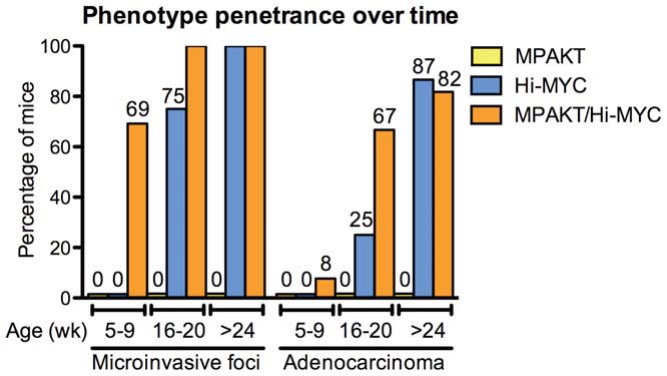
MYC and AKT cooperate to accelerate progression to microinvasion and adenocarcinoma in the MPAKT/Hi-MYC model. Progression of a neoplastic phenotype (all prostate lobes) in MPAKT, Hi-MYC and MPAKT/Hi-MYC, defined by presence of mPIN, stromal remodeling typical of microinvasion, and development of adenocarcinoma. Prostates assessed at 5–9 weeks (13–14 transgenics/group), 16–20 weeks (4–6/group), and 6 months or greater (11–15/group). Trend for progression to microinvasion and to adenocarcinoma, respectively, was significant in Hi-MYC (p<0.0001, p = <0.0001) and MPAKT/Hi-MYC (p = 0.02, p = 0.0002) (Cochran-Armitage trend test). Significant differences (p<0.05, Fisher's exact test) for microinvasion at 5–9 weeks: MPAKT/Hi-MYC vs Hi-MYC (p<0.001), MPAKT/Hi-MYC vs MPAKT (p<0.001); microinvasion at 16–20 weeks: MPAKT/Hi-MYC vs MPAKT (p = 0.002), Hi-MYC vs MPAKT (p = 0.03); microinvasion or adenocarcinoma, respectively at >24 weeks: MPAKT/Hi-MYC vs MPAKT (p = <0.0001, p = <0.0001), Hi-MYC vs MPAKT (p = <0.0001, p = <0.0001). P = 0.06 for adenocarcinoma in MPAKT/Hi-MYC vs Hi-MYC at 16–20 weeks.

Immunohistochemistry for phospho-AKT(Ser473) (pAKT) confirmed AKT activation in MPAKT and, at lower levels, in bigenic MPAKT/Hi-MYC mice (Figs. 3B, S3A, S4). Similarly, immunohistochemical staining of MYC confirmed expression of the *MYC* transgene in Hi-MYC and MPAKT/Hi-MYC mice (Figs. 3B, S3A). Bigenic animals expressed lower levels of transgenic mRNA than single transgenic mice (Fig. S3B). By 5–9 weeks, all three strains (MPAKT, Hi-MYC and bigenic MPAKT/Hi-MYC mice) had mPIN as expected (largely affecting VP in MPAKT mice, DP and LP in Hi-MYC mice, but DP, LP and VP in bigenic mice) (Fig. 3A). Although the growth pattern of mPIN lesions in Hi-MYC and MPAKT/Hi-MYC mice were similar and often cribriform, nuclear atypia was more pronounced in bigenic mice (Fig. S5). At this early time-point, the key distinguishing feature in MPAKT/Hi-MYC mice was significant stromal proliferation, inflammation and remodeling in VP and LP with disruption of the basement membrane and smooth muscle layer surrounding glands affected by mPIN, and presence of epithelial cell clusters within adjacent stroma (Figs. 3A, 4). This stromal remodeling phenotype was further investigated by immunohistochemistry for smooth muscle actin (SMA) and collagen IV, which revealed progressive disruption and loss of the smooth muscle layer and basal laminae in focal points around the proliferative glands suggesting early microinvasion in ∼70% of bigenic mice (Figs. 3C, 4, S6) [33]. In summary, the histopathological features of mPIN lesions in the bigenic mice were similar to those of Hi-MYC mice; however, the stromal remodeling and inflammation, particularly severe in the VP and LP, together with the nuclear atypia of proliferative cells within areas of mPIN, were unique features of the bigenic mice (Figs. 3, 4, S5, S6).

Progression to adenocarcinoma was accelerated in the MPAKT/Hi-MYC model with evidence of invasion in 8% of mice at 5–9 weeks, and in 67% mice at 16–20 weeks, compared respectively with 0% and 25% of Hi-MYC mice (Figs. 3A, 3D, 4; sample size precluded statistical significance). Of note is that pAKT expression was occasionally evident in populations of cells near the invasive regions (Fig. S4). In more advanced disease beyond 6 months of age, the acceleration in disease progression conferred by AKT activation in presence of MYC overexpression was no longer evident (Fig. 4) (due to the highly penetrant MYC alone phenotype), although the unique stromal reaction persisted in the bigenic phenotype.

### The MPAKT/Hi-MYC prostate lesions are accompanied by infiltration of immune cells

**Figure 5:**
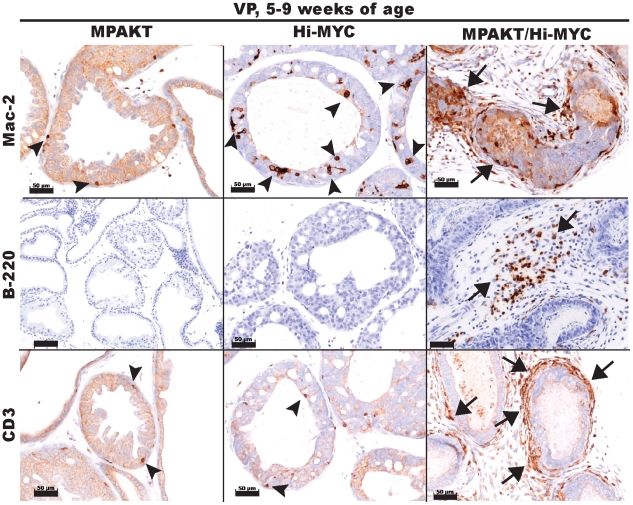
The MPAKT/Hi-MYC phenotype is characterized by stromal inflammation. IHC of VP from mice aged 5–9 weeks, using antibodies to differentiate macrophages (Mac-2), B-lymphocytes (B-220) or T-lymphocytes (CD3). Note periglandular stromal infiltration (*arrows*) in areas of mPIN, unique to MPAKT/Hi-MYC (*right panels*) as compared to MPAKT or Hi-MYC. A few macrophages (*upper panels*) and T-lymphocytes (*lower panels*) are present within glandular epithelium in mPIN from MPAKT and Hi-MYC (*arrowheads*). Scale-bars: 50 µm.

The tumor microenvironment can significantly influence tumorigenesis, and cells from the stromal compartment such as fibroblasts and inflammatory cells can exert effects on adjacent epithelial cells via paracrine signals and extracellular matrix components [34]. To characterize the intense stromal remodeling and inflammatory infiltrate surrounding mPIN and prostate tumors in MPAKT/Hi-MYC mice, we performed immunohistochemistry for T-lymphocytes (CD-3), B-lymphocytes (B220) and macrophages (Mac-2) on prostate tissues from mice aged 5-9 weeks (Fig. 5). All three classes of immune cells were present at high concentrations in the stromal infiltrate and in lesser amounts within the epithelial compartment of mPIN lesions and tumors of the MPAKT/Hi-MYC prostates. In contrast, only occasional macrophages and T-cells were found surrounding mPIN lesions in Hi-MYC prostates, and rare or no inflammatory cells were noted in MPAKT or WT prostates. Thus, the unique stromal remodeling and early invasive phenotype resulting from cooperation between *AKT1* and *MYC* in the mouse prostate is associated with an infiltration of T- and B-lymphocytes, as well as macrophages.

### AKT does not rescue MYC-induced apoptosis in the prostate

To explore the cellular mechanism of AKT-MYC cooperativity, we examined the prostates of bigenic mice and their littermates, using markers of proliferation and apoptosis. As expected [28], elevated levels of both proliferation (Ki67 staining) and apoptosis (measured by TUNEL staining of DNA fragments) were seen in Hi-MYC mPIN lesions (Fig. S7), consistent with the well-established fact that MYC can induce both cell-proliferation and apoptosis [22]. In contrast, Ki67 and TUNEL ratios were only modestly elevated in MPAKT mice compared with WT (Fig. S7) [14], [29]. Ki67 staining in VP and LP of MPAKT/Hi-MYC was comparable to Hi-MYC littermates, with highest proliferative rates occurring in mPIN lesions. Previous reports using different model systems and tissue-types have suggested PI3K-pathway activation can rescue the proapoptotic phenotype of MYC overexpression [35], providing a potential mechanism for cooperativity. However, apoptotic rates remained high in mPIN lesions of MPAKT/Hi-MYC mice and were not obviously different from Hi-MYC littermates.

### Transgenic MYC expression abrogates the mTOR-dependence of the AKT-induced mPIN phenotype

**Figure 6:**
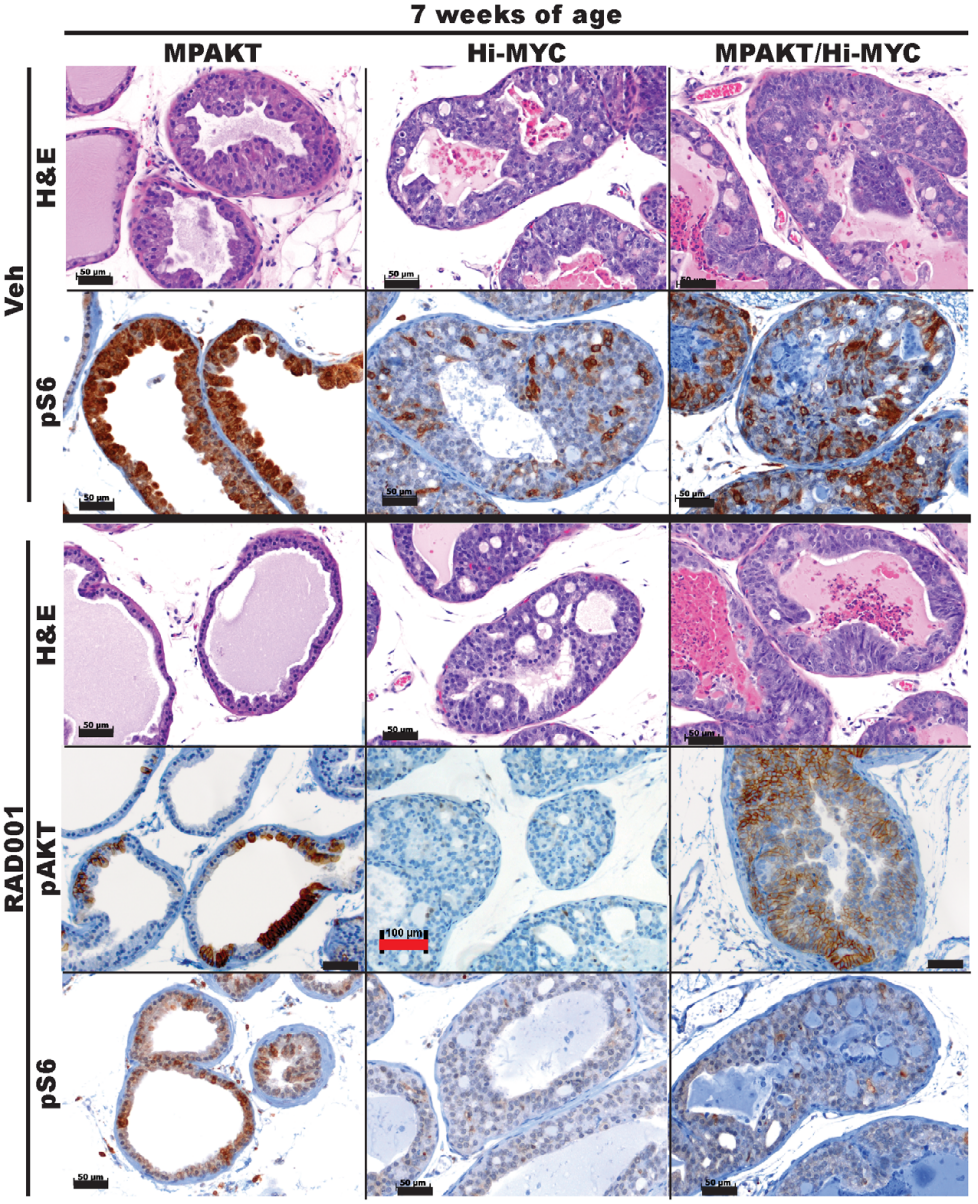
Dependence of the MPAKT phenotype on mTOR is abrogated by MYC expression, and attenuated with age. Prostates from mice aged 7 weeks, treated for 2 weeks with either vehicle or 10 mg/kg RAD001. *H&E panels:* RAD001 treatment fully reverts the mPIN phenotype in VP of 7-week-old MPAKT (n = 4–5/treatment group) - note reversion of nuclear atypia and re-polarization of basal nuclei. RAD001 treatment did not revert LP lesions in Hi-MYC (n = 6/treatment group) and in MPAKT/Hi-MYC (n = 5–6/treatment group) (H&E). *IHC panels:* Patchy pAKT expression remains detectable in reverted lesions of MPAKT mice, and in MPAKT/Hi-MYC, despite RAD001 treatment (see Fig. 3B for pAKT staining in vehicle-treated mice). pS6 is strongly expressed in areas of mPIN in VP of vehicle-treated MPAKT and reduced by RAD001 treatment. pS6 staining in LP of vehicle-treated Hi-MYC and MPAKT/Hi-MYC is patchy in mPIN. RAD001 treatment lowers but does not eliminate pS6 staining in Hi-MYC and MPAKT/Hi-MYC. Scale-bars: 50 µm (black), 100 µm (red).

The AKT-induced mPIN phenotype in young MPAKT mice is dependent on mTOR [14]. We confirmed this in a cohort of 5-week-old MPAKT mice treated with RAD001 or placebo (4-5 mice/treatment group) for 2 weeks (Fig. 6, left panels). As expected, mPIN lesions in a cohort of 5-week-old Hi-MYC mice did not revert after two weeks of RAD001 treatment and were histologically indistinguishable from the lesions in control mice (Fig. 6, center panels) confirming that mPIN in Hi-MYC mice does not depend on mTOR signaling. We next examined the mTOR dependence of mPIN lesions in bigenic MPAKT/Hi-MYC mice by treatment of 5-week-old animals with either RAD001 or placebo for 2 weeks. No reversion of the mPIN phenotype upon RAD001 treatment was observed in the VP and LP of the MPAKT/Hi-MYC mice, and the lesions were identical to those of vehicle-treated mice (Fig. 6, right panels).

To confirm that mTOR was inhibited in RAD001-treated mice, we examined the phosphorylation status of the downstream mTOR substrate ribosomal-S6 protein by immunohistochemistry with a widely-used phosphospecific antibody to Ser235/236 (pS6). In all vehicle-treated MPAKT mice, pS6 in the regions of mPIN was similarly high, and treatment with RAD001 led to dramatically reduced pS6 staining (Fig. 6), indicating that RAD001 effectively inhibited mTOR. pAKT expression was retained, confirming continued transgene expression (Fig. 6). pS6 staining was also decreased by RAD001 treatment in MPAKT/Hi-MYC and Hi-MYC mice, with some tissues showing residual weak pS6 staining (Fig. 6). S235/236 of S6 is also the site for phosphorylation by p90 ribosomal kinase (RSK), raising the possibility of mTORC1-independent (therefore RAD001-resistant) phosphorylation of S6 [36].

In summary, mPIN lesions in young MPAKT mice were fully reverted upon RAD001-treatment; however, mPIN lesions in Hi-MYC and MPAKT/Hi-MYC bigenic mice did not respond to RAD001 despite effective mTORC1 inhibition. We conclude that transgenic MYC expression is sufficient to override the mTOR dependence of lesions arising from constitutive AKT activation. RAD001 treatment did not affect intensity or composition of the inflammatory infiltrate in prostates of bigenic mice.

**Figure 7:**
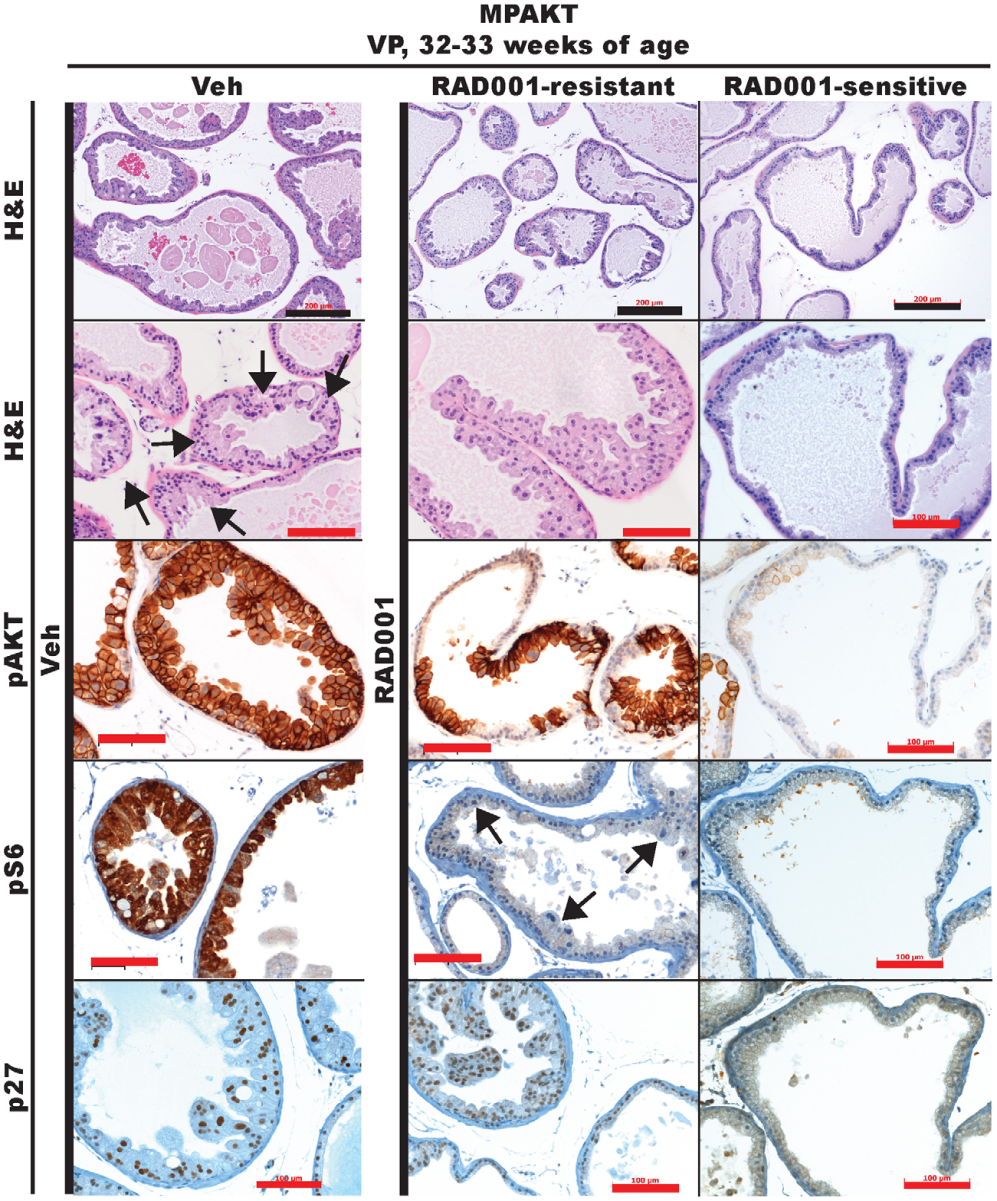
Dependence of the MPAKT phenotype on mTOR is abrogated by MYC expression, and attenuated with age. Prostates from mice aged 32–33 weeks, treated for 2 weeks with either vehicle or 10 mg/kg RAD001. RAD001 treatment only variably reverts mPIN phenotype in VP of 32–33-week-old MPAKT (H&E: *upper panels*: low-magnification, *lower panels*: high-magnification). mPIN lesions are preserved in vehicle-treated and RAD001-resistant examples (diffuse in H&E, patchy in pS6 representative images (*arrows*). pS6 is strongly expressed in areas of mPIN in VP of vehicle-treated MPAKT, and reduced by RAD001-treatment. (n = 6–7/treatment group; only 2 of 7 RAD001-treated mice responded). Scale-bars: 200 µm (balck), 100 µm (red).

The mTOR dependence of the activated AKT-driven mPIN phenotype has been demonstrated only in young MPAKT mice [14]. Having demonstrated that MYC can rescue the mTOR dependence of AKT-driven mPIN lesions, we asked if the mPIN lesions of older MPAKT mice would remain dependent on mTOR, or whether additional genetic lesions potentially accumulated with aging might render the prostate lesions insensitive to RAD001 treatment. In contrast to young MPAKT mice, the response of older MPAKT mice (28–32weeks-old) to mTOR inhibition was incomplete and variable (Figs. 7, S8). Of seven mice treated with RAD001 for two weeks, five had residual mPIN, whereas two had no evidence of mPIN. As expected, mPIN was detected in the VP of all 6 placebo-treated mice. pAKT was expressed in mPIN of vehicle-treated MPAKT mice and in both RAD001-sensitive and RAD001-resistant mice, whereas loss of pS6 staining in all RAD001-treated animals confirmed mTOR inhibition (Fig. 7). Strong p27 expression, a documented marker of mPIN in MPAKT mice [37], was observed in mPIN of the vehicle-treated and RAD001-resistant MPAKT mice, but absent in WT animals and in the reverted lesions of RAD001-sensitive mice, providing additional evidence for RAD001-resistance (Fig. 7). Therefore, the mPIN phenotype of MPAKT mice becomes progressively independent of mTOR with age.

**Figure 8:**
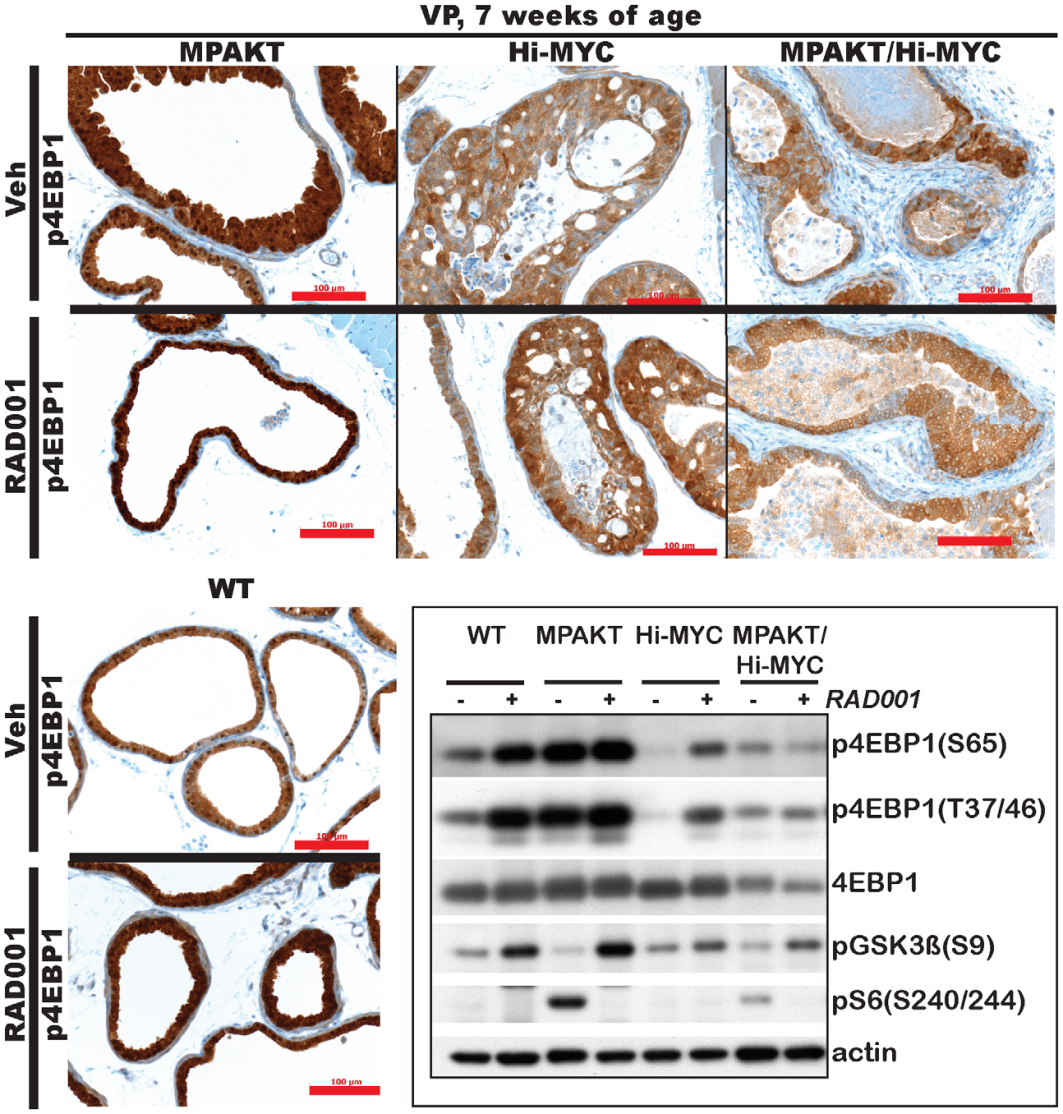
RAD001 increases p4EBP1 expression across genotypes, and decreases TUNEL in MPAKT/Hi-MYC and Hi-MYC tumors. Prostates from mice aged 7-weeks, treated with either vehicle or 10 mg/kg RAD001. *Upper panels*, *lower left panel*: IHC of VP from 7-week-old mice. 14 d RAD001 treatment did not block p4EBP1 expression in prostates of any genotype (n = 4–6/treatment group, /genotype). Scale-bars: 100 µm. *Lower right panel*: Cropped immunoblots after SDS-PAGE gel electrophoresis of protein lysates from VP of 7-week-old mice treated 2 d with vehicle or RAD001 (full-length blots in Fig. S9). RAD001 did not lower (instead increased) p4EBP1 expression, despite successful inhibition of mTORC1, indicated by decreased expression of downstream target pS6 and feedback upregulation of pGSK3β levels.

We next asked whether 4EBP1, an mTORC1 target, plays a role in mediating the sensitivity to RAD001 in MPAKT mice, and the RAD001-resistance in the Hi-MYC and MPAKT/Hi-MYC models, as proposed by a study that used genetically engineered prostate epithelial cells to examine the affect of MYC expression on rapamycin sensitivity [23]. Surprisingly, immunohistochemical assessment of 4EBP1 phosphorylation in the VP of mice aged 7-weeks showed no decline in p4EBP1 levels in MPAKT mice following 2 weeks of RAD001 treatment (Fig. 8), despite clear histologic regression of mPIN lesions (Figs. 6, 8). Similarly, expression of p4EBP1 in wild type, Hi-MYC and MPAKT/Hi-MYC mice was either unchanged or slightly *increased* by RAD001 treatment (Fig. 8). We confirmed this result by immunoblot of protein lysates from isolated ventral prostates, and verified the increased 4EBP1 phosphorylation in the VP of RAD001-treated mice, independent of total 4EBP1 expression (Fig. 8, S9). Abrogation of pS6 expression along with increased glycogen synthase kinase-3β (GSK3β) phosphorylation (a measure of AKT activation due to relief of mTORC1-mediated feedback inhibition of AKT) confirmed successful inhibition of mTOR (Figs. 8, S9). Therefore 4EBP1 phosphorylation in WT, MPAKT, Hi-MYC and MPAKT/Hi-MYC mice is not uniquely dependent on mTOR and cannot explain resistance to mTOR inhibition.

**Figure 9:**
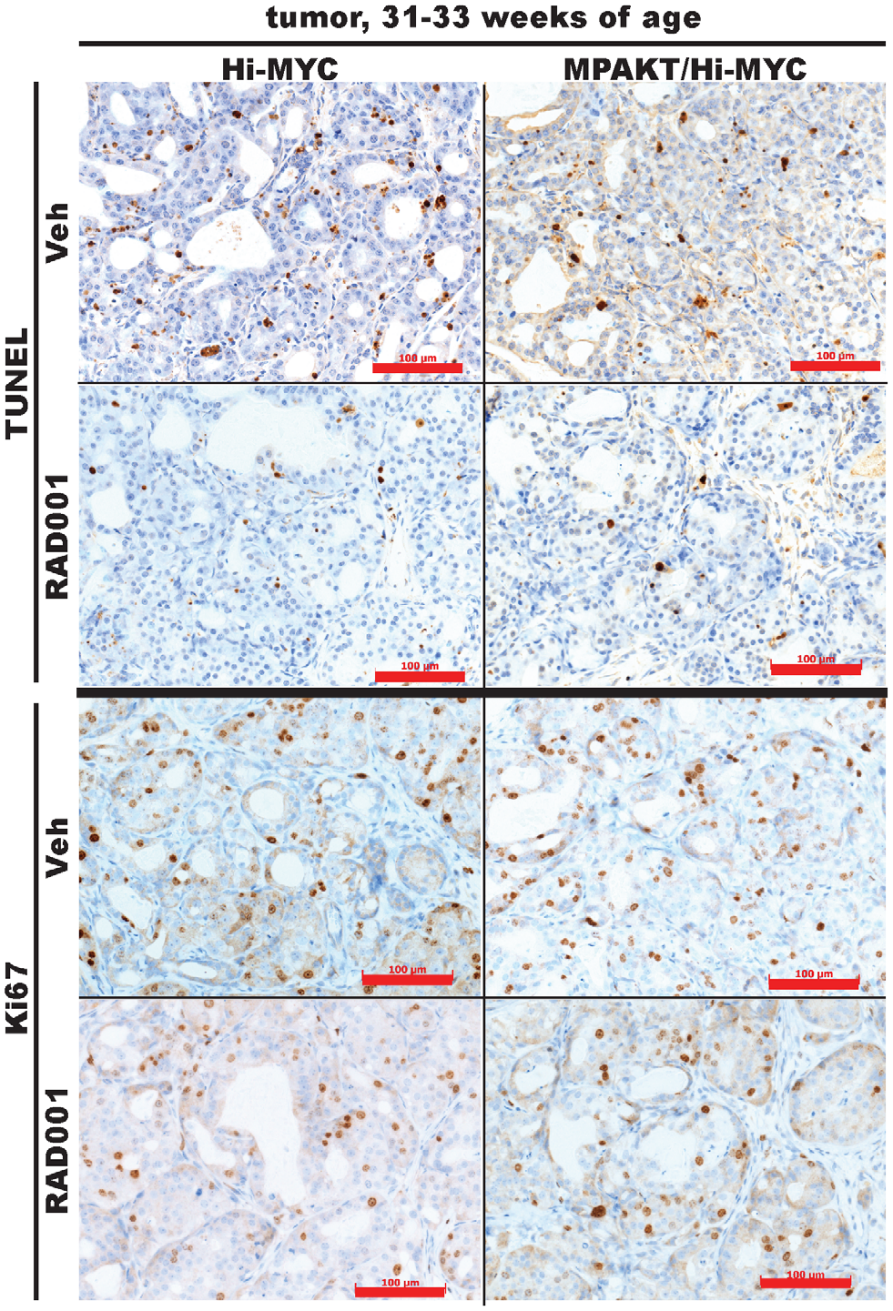
RAD001 increases p4EBP1 expression across genotypes, and decreases TUNEL in MPAKT/Hi-MYC and Hi-MYC tumors. Prostates from mice aged 31–33 weeks, treated with either vehicle or 10 mg/kg RAD001. Representative TUNEL and Ki67 IHC-staining of prostate tumors from 31–33week-old Hi-MYC and MPAKT/Hi-MYC. 14 d RAD001 treatment reduces TUNEL-staining, indicative of reduced apoptosis, but has minimal effect on the Ki67 proliferative-marker. Images representative of all Hi-MYC (4/4 vehicle-treated, 5/5 RAD001-treated) and MPAKT/Hi-MYC (5/5 vehicle-treated, 5/5 RAD001-treated) examined. Scale-bars: 100 µm.

MYC expression may confer resistance to rapamycin by disrupting the balance between proliferation and apoptosis or senescence. Interestingly, prostate tumors from Hi-MYC (n = 5) and MPAKT/Hi-MYC (n = 5) mice all showed *reduced* TUNEL staining after 14 days of RAD001 treatment compared to prostates from vehicle-treated animals (n = 4–5/genotype) (Fig. 9, S10). The Ki67 staining in the same tissues was unaffected by RAD001 treatment (Fig. 9, S10). Therefore, MYC expression does not simply confer resistance to mTOR inhibition. The reduction in apoptosis may, in fact, reveal paradoxical effects of mTOR inhibitors on tumor progression.

## Discussion

PI3K-pathway upregulation in primary and metastatic prostate cancers provides the rationale for clinical evaluation of PI3K-pathway inhibitors (including rapalogs). Here we demonstrate a statistically significant co-occurrence of *MYC* amplification and PI3K-pathway disruption in 194 human prostate tumors, including 37 metastatic tumors. To investigate the potential functional interaction between the MYC and PI3K-pathways in the prostate, we first generated a PTEN^pc−/−^/Hi-MYC bigenic mouse that confirmed a prior model of cooperativity between these two pathways [24]. Next, to further investigate the role of PI3K downstream mediators in the interaction with *MYC*, we crossbred previously characterized mice expressing activated human AKT1 (MPAKT model) [14], [29] and human MYC (Hi-MYC model) [28]. In the resultant MPAKT/Hi-MYC model, AKT1 and MYC are expressed together in the prostate, recapitulating the co-incidence of the genetic lesions in human prostate tumor samples.

The prostate glands of MPAKT/Hi-MYC mice are characterized by significant stromal reaction and infiltration of B- and T-lymphocytes, as well as macrophages early in development of mPIN and persisting throughout tumorigenesis. This inflammatory response is of particular interest because of possible roles for the immune system in tumor growth regulation. In the prostate, inflammation is commonly observed in cancer precursor lesions [34], [38]. In addition, recent work has implicated infiltrating T_H_17 and/or T_reg_ (FoxP3^+^) T-cells in development or progression of human prostate cancer [39]. Cytokines can confer survival to tumor cells in xenografts derived from the Hi-MYC model, facilitating prostate cancer progression [40]. Since it remains unclear to what extent the inflammatory cells in human samples play an active versus bystander role in cancer progression or suppression, the MPAKT/Hi-MYC model may help address this question. Indeed, genetically engineered mouse models of other tumor types (breast, pancreatic islet cells, etc) have firmly established both tumor-promoting and -suppressive actions for distinct subsets of inflammatory cells [41].

Due to growing interest in evaluating PI3K-pathway inhibitors in prostate cancer patients, we explored the activity of the rapamycin analog RAD001 in the MPAKT/Hi-MYC model. In contrast to the exquisite sensitivity of young MPAKT mice to this compound [14], MPAKT/Hi-MYC as well as older MPAKT mice were completely or partially resistant, respectively. The mechanism of resistance remains to be determined but we can likely exclude pharmacologic explanations such as incomplete target inhibition. Because recent evidence suggests perturbations in levels of the eukaryotic elongation factor 4E (eIF-4E) or its inhibitor 4EBP1, a translational regulator acting downstream of AKT and mTOR, could mediate resistance [42] [43] [23], we considered this as a potential mechanism for RAD001-resistance in the MPAKT/Hi-MYC mice. However, bioinformatic mining of published transcriptome data [28], [29] revealed no significant changes in levels of 4EBP1 or eIF-4E in prostate tissues from Hi-MYC or MPAKT mice. Furthermore, phosphorylation of 4EBP1 was unimpaired by mTOR inhibition in these mice (but rather *increased*, possibly due to feedback activation of the MEK/ERK-pathway [44]). Thus 4EBP1 is not a predictor of response to rapalog therapy in these mice.

Rapalogs, which selectively inhibit the TORC1 complex, can paradoxically activate AKT through loss of S6 kinase-mediated negative feedback at the level of PI3K [17]. While RAD001 resistance could be theoretically mediated through AKT activation that results from TORC1 blockade, it is difficult to envision why this would occur selectively in the MPAKT/Hi-MYC mice and not in the young MPAKT mice, which are RAD001-sensitive. Indeed, our analysis of phospho-AKT levels in RAD001 treated animals revealed similar effects in both strains. Interestingly, the rapamycin-resistant PrEC cells expressing activated PI3K and MYC were sensitive to the dual PI3K/mTOR inhibitor BEZ235 [23], raising the possibility that reduced AKT activity is critical for response.

Another potential mechanism for rapalog-resistance may be the documented mitigation of cellular senescence upon mTOR inhibition in tumors with activated senescence programs [45]. We observed no consistent changes in expression of the senescence-marker p27 by immunohistochemistry in MPAKT/Hi-MYC and Hi-MYC prostates following RAD001 treatment (not shown); however, we did observe a reduction in TUNEL staining in RAD001-treated tumors. The mechanism of this pro-survival effect of RAD001 treatment in the context of MYC expression could be mediated through relief of mTOR-mediated feedback (eg AKT activation) or other mechanisms requiring further study.

Rapalogs have been explored in pilot studies in prostate cancer, and PI3K and mTORC1/2 kinase inhibitors are now in early-stage clinical trials across tumor types. In this context, our demonstration that MYC overexpression can convert AKT-activated mouse prostate tumors from rapalog-sensitive to rapalog-resistant has implications for clinical studies of PI3K-pathway inhibitors in men whose prostate cancers also harbor increased AKT signaling. As is clear with other tumor types such as glioblastoma and breast cancer, secondary genetic alterations such as PTEN loss can mitigate the response to EGFR or HER2 inhibitors [46], [47]. In light of the relatively disappointing single agent activity of rapalogs in prostate cancer, it may be critical to assess the MYC status of prostate tumors (among several other markers) to guide the interpretation of response data in patients undergoing PI3K inhibitor therapy.

## Supporting Information

Figure S1 ***MYC*** **amplification co-occurs with AKT pathway activation in human prostate tumor samples.** Contingency tables for co-occurrence of *MYC* and PI3K-pathway copy-number alterations (defined by aCGH) from (A) 157 primary and 37 metastatic prostate tumor samples (n  =  194 total), or from (B, C) 37 metastatic samples alone. The tables indicate in red the proportion of tumors with *PIK3CA* amplification or general PI3K pathway gain, that also exhibit *MYC* amplification. (A), (B) The association between multi-copy *MYC* amplification and single- or multi-copy PI3K-pathway alterations is shown. (C) The association between single- or multi-copy *MYC* gain and PI3K pathway alterations is shown. The statistical significance of each association is reported as a P-value determined by 2-tailed Fisher's exact test.
(TIF)

Figure S2 ***MYC*** **amplification co-occurs with AKT pathway activation in human prostate tumor samples.** Graphic representation of contingency tables (Fig. 1, Fig. S1) for co-occurrence of *MYC* and PI3K pathway copy number alterations (as defined by aCGH in Methods) from (A) 157 primary and 37 metastatic prostate tumor samples (n  =  194 total), or from (B) 37 metastatic samples alone. The graphs indicate the proportion of tumors with *PIK3CA* amplification or general PI3K pathway gain, that also exhibit *MYC* amplification. The statistical significance of each association is reported as a P-value determined by 2-tailed Fisher's exact test.
(TIF)

Figure S3 **The AKT and MYC transgenes are expressed in prostates of bigenic MPAKT/Hi-MYC mice, albeit at lower levels than in the single transgenic mice.** (A) Immunohistochemistry using antibodies for pAKT (Ser473) and MYC on ventral prostates from mice aged 30–33 weeks. Note the high levels of pAKT membrane staining associated with regions of mPIN in MPAKT/Hi-MYC mice. pAKT staining is absent in Hi-MYC mice. Nuclear MYC staining is evident in Hi-MYC and MPAKT/Hi-MYC, but absent in MPAKT mice. Scale-bars: 50 µm (black), 30 µm (red). (B) qRT-PCR analysis of the myr-HA-*AKT1* and *MYC* transgenes in prostates from 7 week-old MPAKT, MPAKT/Hi-MYC and Hi-MYC mice (normalized to actin mRNA, mean ± SD, n = 6 prostates per genotype, run in triplicate). P < 0.01 (determined by two-way ANOVA with Bonferroni post-test) for Tg-AKT expression in MPAKT/Hi-MYC vs MPAKT.
(TIF)

Figure S4 **pAKT is expressed in cells near areas of invasion in MPAKT/Hi-MYC mice.** Invasive area in lateral prostate of MPAKT/Hi-MYC mouse aged 21 weeks (upper panels: hematoxylin & eosin). Lower panels: Immunohistochemistry using an antibody for pAKT (Ser473) indicates lower but detectable pAKT expression in tumor cells compared to PIN lesions. Scale bars: 200 µm (black), 100 µm (red).
(TIF)

Figure S5 **Prostate epithelial cells display a higher degree of nuclear atypia in PIN lesions from MPAKT/Hi-MYC mice than from Hi-MYC mice.** mPIN lesions in 8-week-old lateral prostates from Hi-MYC and MPAKT/Hi-MYC mice, indicating a greater degree of nuclear atypia in the MPAKT/Hi-MYC cells with larger nuclei and more open chromatin (H&E). Scale bars: 20 µm.
(TIF)

Figure S6 **The MPAKT/Hi-MYC ventral prostate displays areas of stromal remodeling characteristic of microinvasive foci.** Immunohistochemistry for smooth muscle actin and collagen IV providing additional examples (as in Fig. 3C) of disrupted and absent smooth muscle stroma and collagen IV surrounding glands affected by mPIN in the MPAKT/Hi-MYC prostate (*middle and right columns*). Note (*left column*), an example of minimally attenuated smooth muscle sheath and collagen IV around a gland with no evidence of microinvasion. Scale bars: 100 µm.
(TIF)

Figure S7 **The MPAKT/Hi-MYC phenotype is characterized by an increase in cell proliferation and apoptosis compared to MPAKT mice, and the pro-apoptotic effects of MYC are not rescued by AKT expression.** Mouse prostates from animals aged 5–9 weeks were stained with TUNEL or antibody to Ki67. Results were quantified as the mean percentage (± SD) of positive cells, counting at least 300 cells from representative areas (ventral prostate for most mice, and mPIN lesions where present) from each of 9 mice per transgenic genotype and 4 wild-type mice. P < 0.05 (determined by an untailed t-test with two-tail p-value) for MPAKT/Hi-MYC vs MPAKT, MPAKT/Hi-MYC vs WT, Hi-MYC vs MPAKT, Hi-MYC vs WT.
(TIF)

Figure S8 **mPIN lesions of older MPAKT mouse prostates are variably resistant to RAD001.** Additional examples (as in Figure 7) showing that 14d RAD001 treatment only variably reverts the mPIN phenotype in ventral prostates of 32–33 week-old MPAKT mice (H&E). Note that mPIN lesions are preserved in the RAD001-resistant examples, whereas the RAD001-sensitive MPAKT example shows normal glands similar to that of a vehicle-treated wild-type mouse. Note also that even in hyperplastic areas of the wild-type glands, there is no nuclear atypia or cellular hypertrophy (6–7 mice per treatment group; only 2 of 7 RAD001-treated MPAKT mice responded). Scale bars: 200 µm (black), 100 µm (red).
(TIF)

Figure S9 **Uncropped immunoblots.** Immunoblots from which portions were cropped for display in Figure 8. Immunoblots were prepared from 3 separate gels with matched loading of identical samples, after SDS-PAGE electrophoresis of lysates from ventral prostates of 7-week-old mice treated for 2 days with vehicle or RAD001. PVDF membranes were cut horizontally prior to probing with primary antibodies for p4EBP1 (Ser65), p4EBP1 (Thr37/46), total 4EBP1, pGSK3β (Ser9), pS6 (Ser240/244), and β-actin to control for protein loading. RAD001 did not lower (but instead increased) p4EBP1 expression, despite successful inhibition of mTORC1 as indicated by decreased expression of downstream target pS6 and feedback upregulation of pGSK3β levels.
(TIF)

Figure S10 **Tumors in prostates of RAD001-treated Hi-MYC and MPAKT/Hi-MYC mice display lower levels of apoptosis and similar levels of proliferation compared with vehicle-treated mice.** Lower magnification views of images displayed in Figure 9. Representative TUNEL and immunohistochemical staining using an antibody for Ki67, of prostate tumors from Hi-MYC and MPAKT/Hi-MYC mice aged 31–33 weeks. 14d RAD001 treatment reduces TUNEL staining in tumors, indicative of reduced apoptosis, but has minimal effect on the Ki67 proliferative marker. Images are representative of all HiMYC mice (4/4 vehicle-treated, 5/5 RAD001-treated) and MPAKT/Hi-MYC mice (5/5 vehicle-treated, 5/5 RAD001-treated) examined. Scale bars: 200 µm (black), 500 µm (red).
(TIF)

Table S1 **Primary copy number alternation data from array CGH analysis.**
(DOC)

Text S1 **Supplemental Materials and Methods.**
(PDF)
