# Supplementary figures and images for: MYC Cooperates with AKT in Prostate Tumorigenesis and Alters Sensitivity to mTOR Inhibitors
Source: PLoS One. 2011 Mar 4;6(3):e17449. doi: 10.1371/journal.pone.0017449 (PMC3048873; doi:10.1371/journal.pone.0017449)

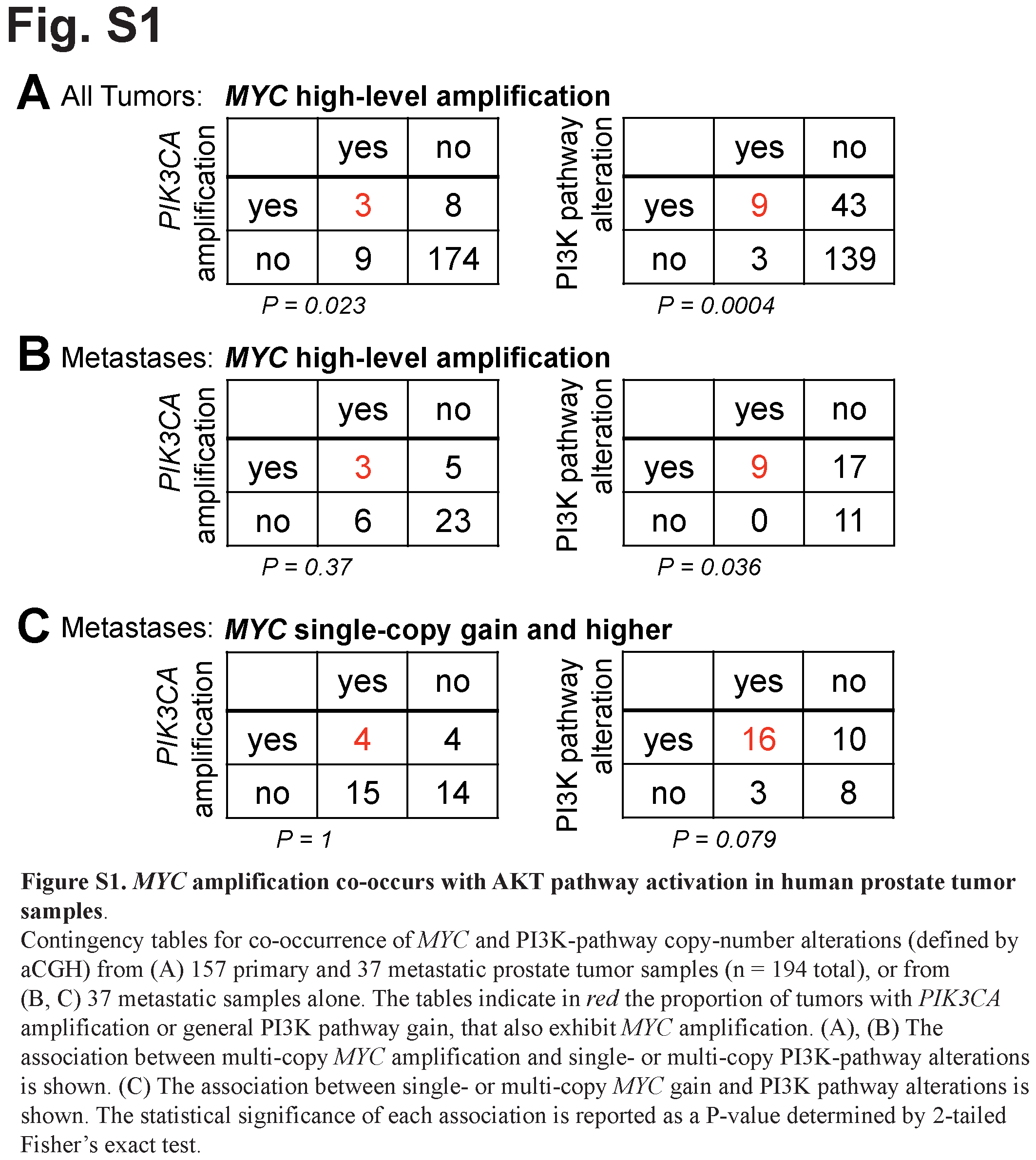

Supplement: Figure S1 — MYC amplification co-occurs with AKT pathway activation in human prostate tumor samples. Contingency tables for co-occurrence of MYC and PI3K-pathway copy-number alterations (defined by aCGH) from (A) 157 primary and 37 metastatic prostate tumor samples (n = 194 total), or from (B, C) 37 metastatic samples alone. The tables indicate in red the proportion of tumors with PIK3CA amplification or general PI3K pathway gain, that also exhibit MYC amplification. (A), (B) The association between multi-copy MYC amplification and single- or multi-copy PI3K-pathway alterations is shown. (C) The association between single- or multi-copy MYC gain and PI3K pathway alterations is shown. The statistical significance of each association is reported as a P-value determined by 2-tailed Fisher's exact test. (TIF) [file pone.0017449.s001.tif]

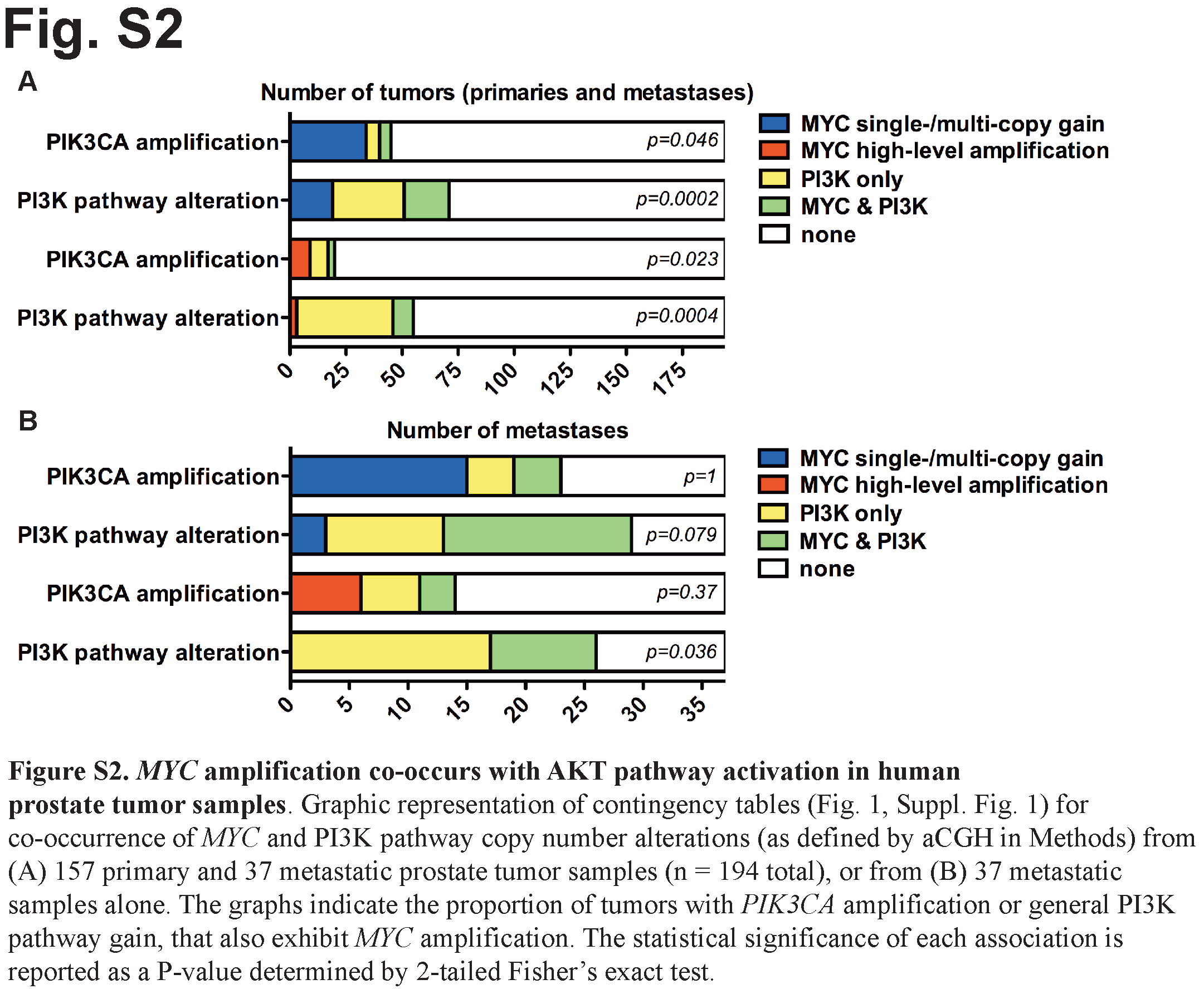

Supplement: Figure S2 — MYC amplification co-occurs with AKT pathway activation in human prostate tumor samples. Graphic representation of contingency tables (Fig. 1, Fig. S1) for co-occurrence of MYC and PI3K pathway copy number alterations (as defined by aCGH in Methods) from (A) 157 primary and 37 metastatic prostate tumor samples (n = 194 total), or from (B) 37 metastatic samples alone. The graphs indicate the proportion of tumors with PIK3CA amplification or general PI3K pathway gain, that also exhibit MYC amplification. The statistical significance of each association is reported as a P-value determined by 2-tailed Fisher's exact test. (TIF) [file pone.0017449.s002.tif]

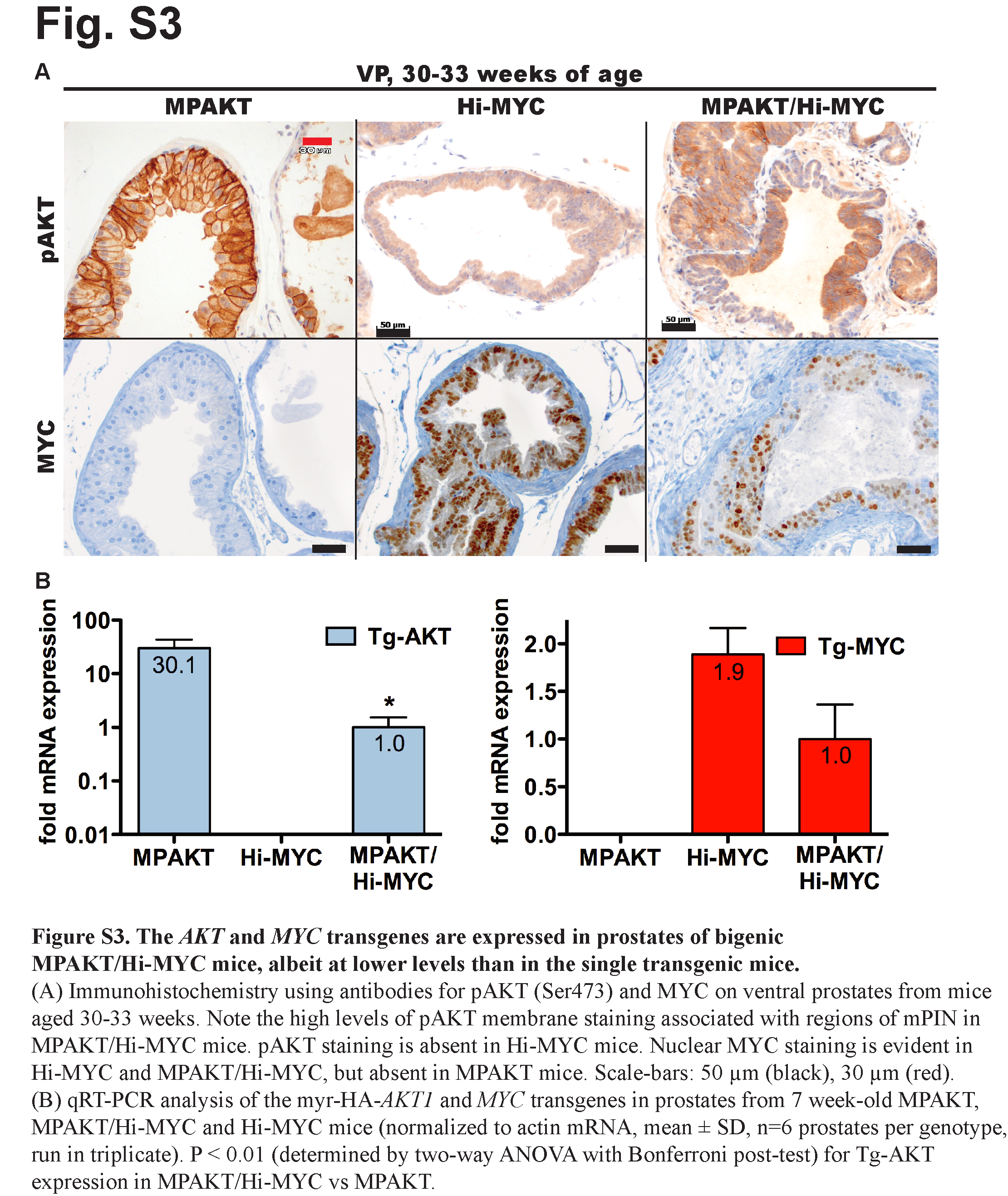

Supplement: Figure S3 — The AKT and MYC transgenes are expressed in prostates of bigenic MPAKT/Hi-MYC mice, albeit at lower levels than in the single transgenic mice. (A) Immunohistochemistry using antibodies for pAKT (Ser473) and MYC on ventral prostates from mice aged 30–33 weeks. Note the high levels of pAKT membrane staining associated with regions of mPIN in MPAKT/Hi-MYC mice. pAKT staining is absent in Hi-MYC mice. Nuclear MYC staining is evident in Hi-MYC and MPAKT/Hi-MYC, but absent in MPAKT mice. Scale-bars: 50 µm (black), 30 µm (red). (B) qRT-PCR analysis of the myr-HA-AKT1 and MYC transgenes in prostates from 7 week-old MPAKT, MPAKT/Hi-MYC and Hi-MYC mice (normalized to actin mRNA, mean ± SD, n = 6 prostates per genotype, run in triplicate). P < 0.01 (determined by two-way ANOVA with Bonferroni post-test) for Tg-AKT expression in MPAKT/Hi-MYC vs MPAKT. (TIF) [file pone.0017449.s003.tif]

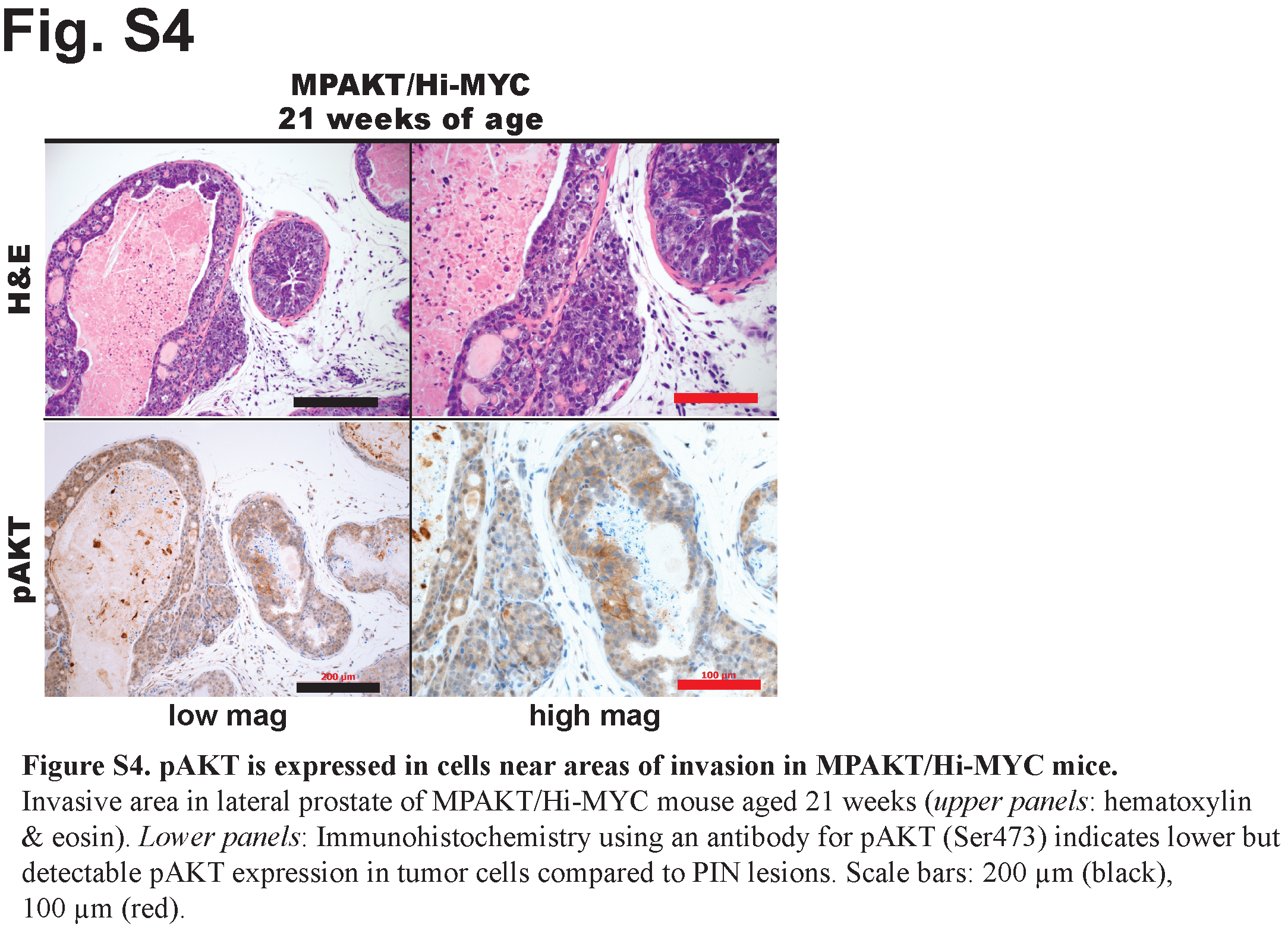

Supplement: Figure S4 — pAKT is expressed in cells near areas of invasion in MPAKT/Hi-MYC mice. Invasive area in lateral prostate of MPAKT/Hi-MYC mouse aged 21 weeks (upper panels: hematoxylin & eosin). Lower panels: Immunohistochemistry using an antibody for pAKT (Ser473) indicates lower but detectable pAKT expression in tumor cells compared to PIN lesions. Scale bars: 200 µm (black), 100 µm (red). (TIF) [file pone.0017449.s004.tif]

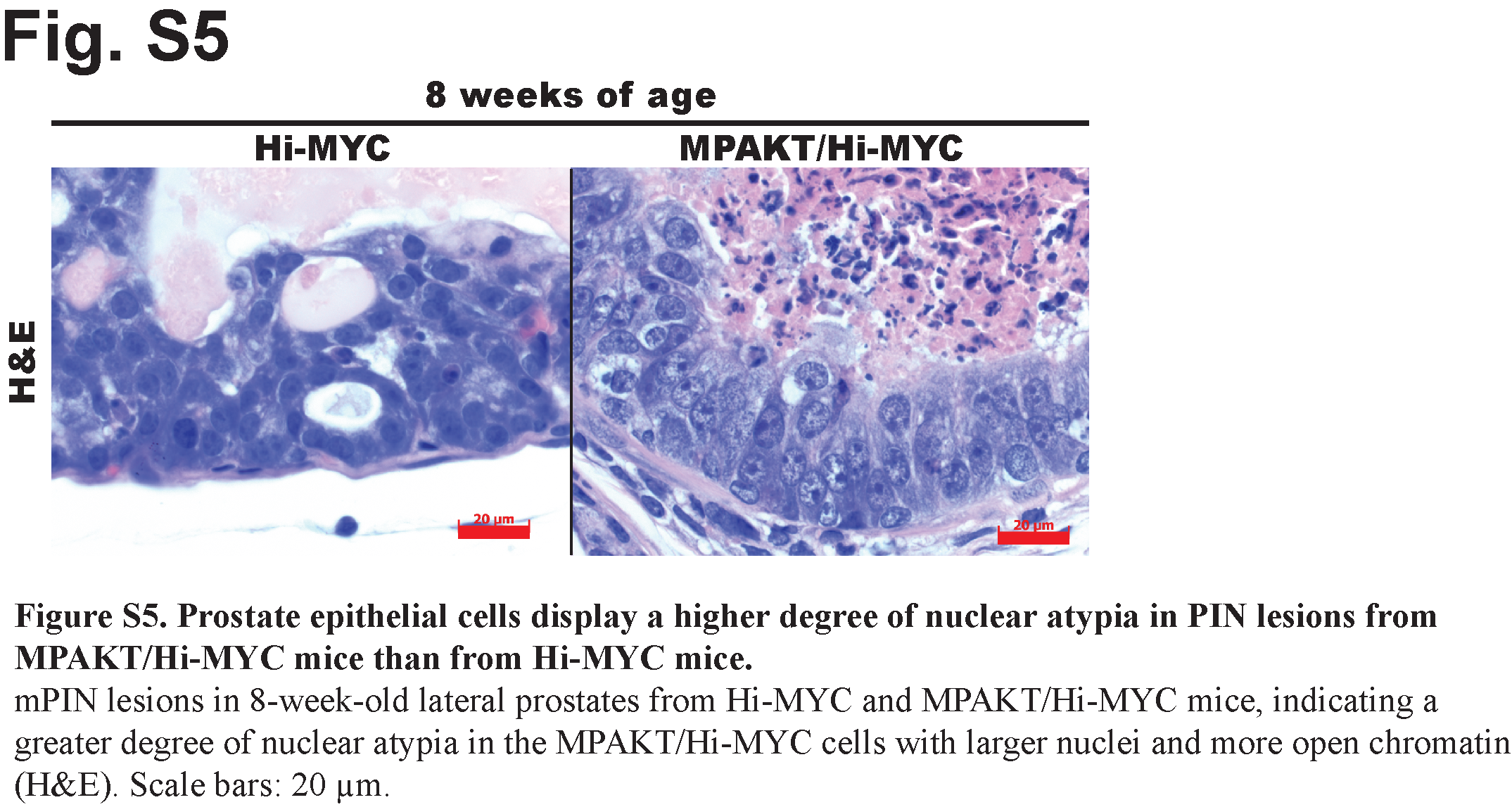

Supplement: Figure S5 — Prostate epithelial cells display a higher degree of nuclear atypia in PIN lesions from MPAKT/Hi-MYC mice than from Hi-MYC mice. mPIN lesions in 8-week-old lateral prostates from Hi-MYC and MPAKT/Hi-MYC mice, indicating a greater degree of nuclear atypia in the MPAKT/Hi-MYC cells with larger nuclei and more open chromatin (H&E). Scale bars: 20 µm. (TIF) [file pone.0017449.s005.tif]

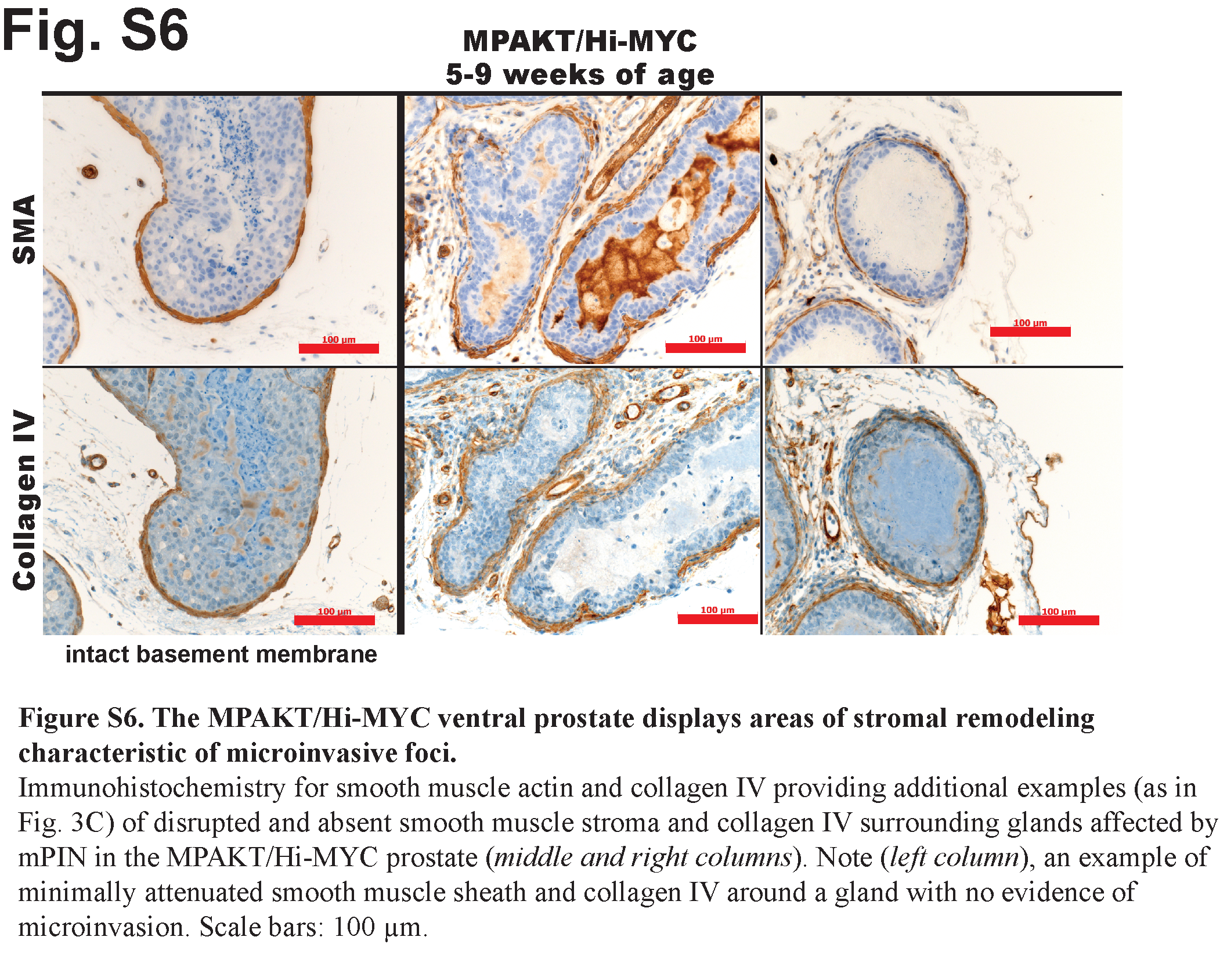

Supplement: Figure S6 — The MPAKT/Hi-MYC ventral prostate displays areas of stromal remodeling characteristic of microinvasive foci. Immunohistochemistry for smooth muscle actin and collagen IV providing additional examples (as in Fig. 3C) of disrupted and absent smooth muscle stroma and collagen IV surrounding glands affected by mPIN in the MPAKT/Hi-MYC prostate (middle and right columns). Note (left column), an example of minimally attenuated smooth muscle sheath and collagen IV around a gland with no evidence of microinvasion. Scale bars: 100 µm. (TIF) [file pone.0017449.s006.tif]

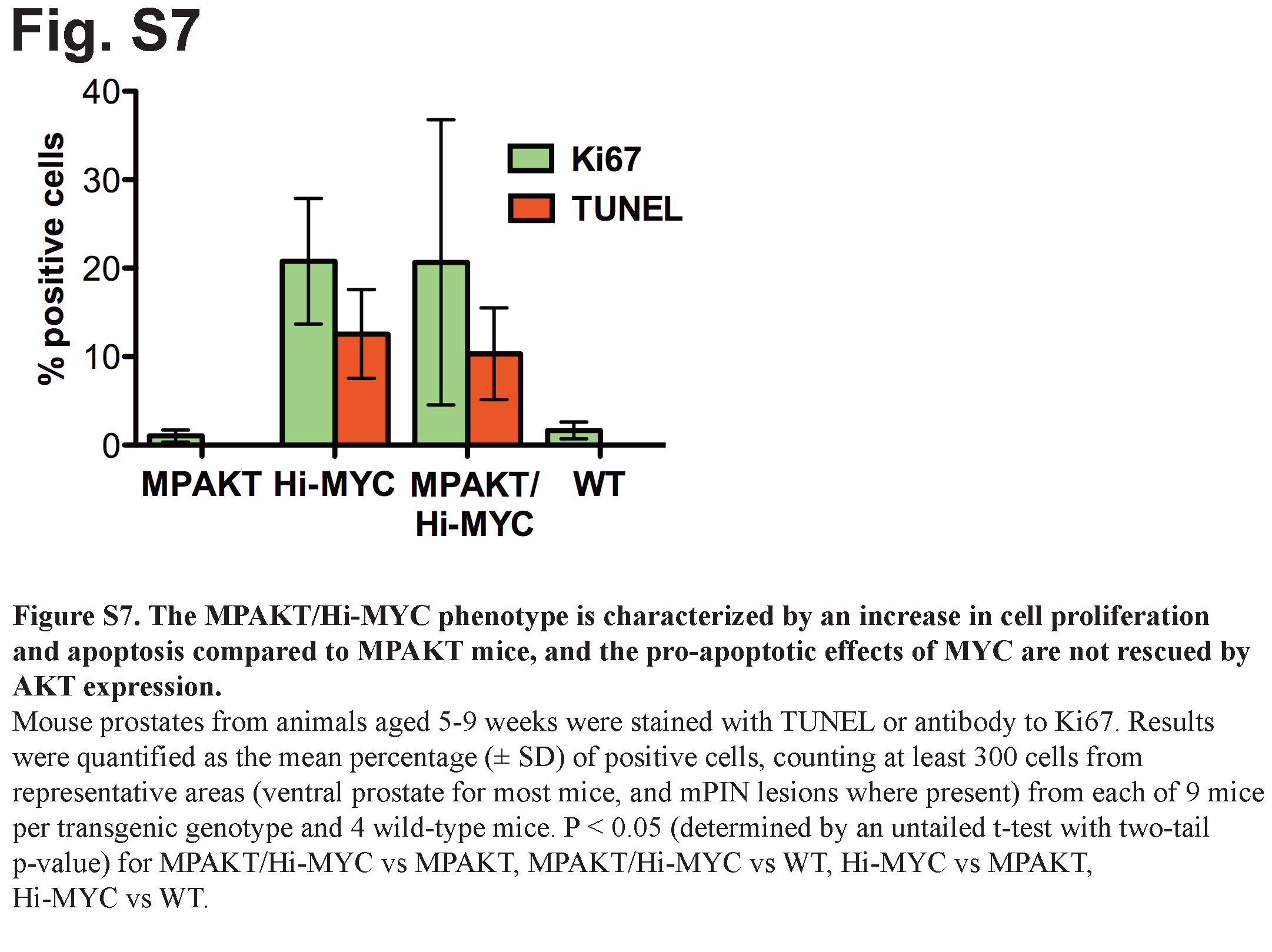

Supplement: Figure S7 — The MPAKT/Hi-MYC phenotype is characterized by an increase in cell proliferation and apoptosis compared to MPAKT mice, and the pro-apoptotic effects of MYC are not rescued by AKT expression. Mouse prostates from animals aged 5–9 weeks were stained with TUNEL or antibody to Ki67. Results were quantified as the mean percentage (± SD) of positive cells, counting at least 300 cells from representative areas (ventral prostate for most mice, and mPIN lesions where present) from each of 9 mice per transgenic genotype and 4 wild-type mice. P < 0.05 (determined by an untailed t-test with two-tail p-value) for MPAKT/Hi-MYC vs MPAKT, MPAKT/Hi-MYC vs WT, Hi-MYC vs MPAKT, Hi-MYC vs WT. (TIF) [file pone.0017449.s007.tif]

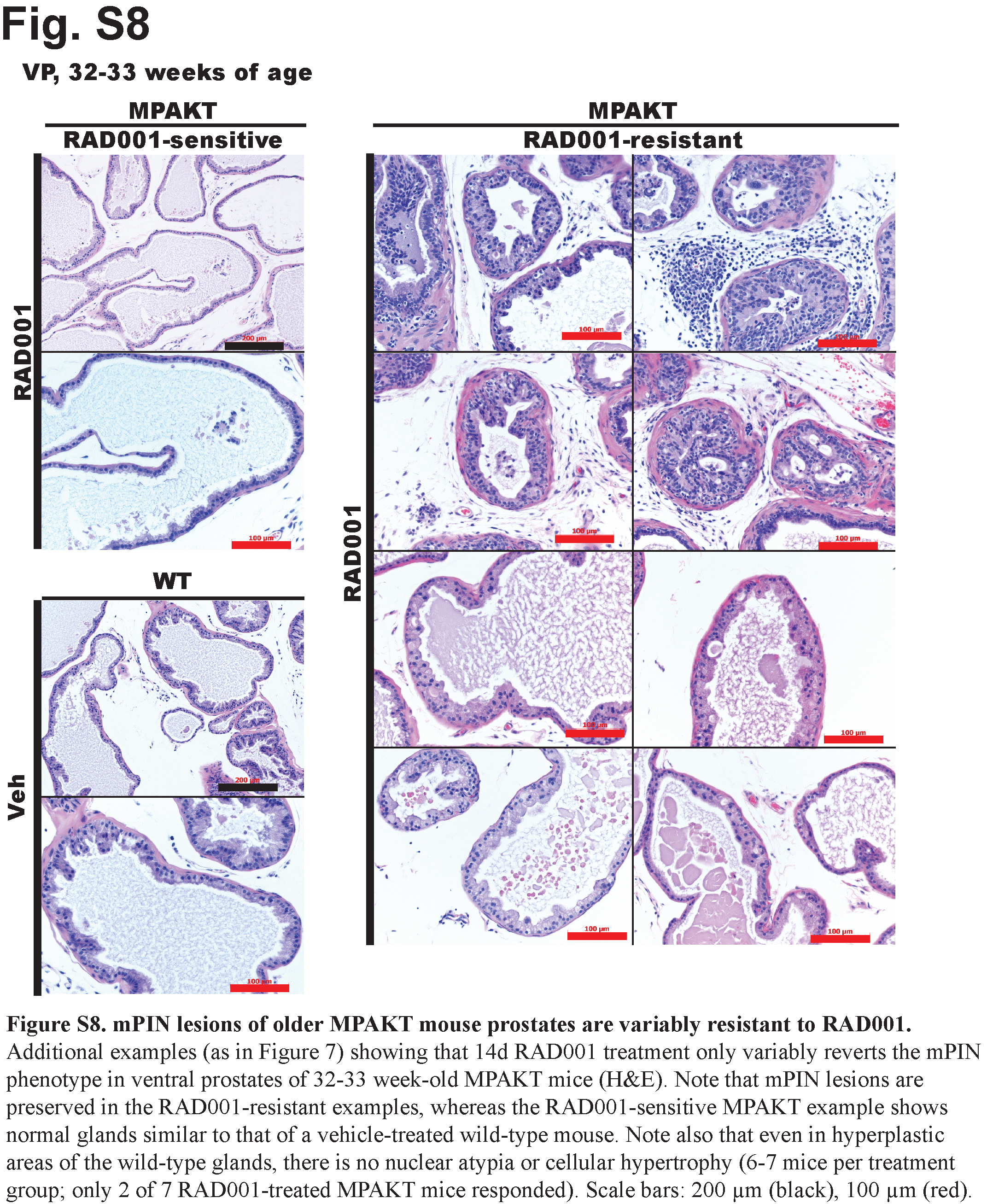

Supplement: Figure S8 — mPIN lesions of older MPAKT mouse prostates are variably resistant to RAD001. Additional examples (as in Figure 7) showing that 14d RAD001 treatment only variably reverts the mPIN phenotype in ventral prostates of 32–33 week-old MPAKT mice (H&E). Note that mPIN lesions are preserved in the RAD001-resistant examples, whereas the RAD001-sensitive MPAKT example shows normal glands similar to that of a vehicle-treated wild-type mouse. Note also that even in hyperplastic areas of the wild-type glands, there is no nuclear atypia or cellular hypertrophy (6–7 mice per treatment group; only 2 of 7 RAD001-treated MPAKT mice responded). Scale bars: 200 µm (black), 100 µm (red). (TIF) [file pone.0017449.s008.tif]

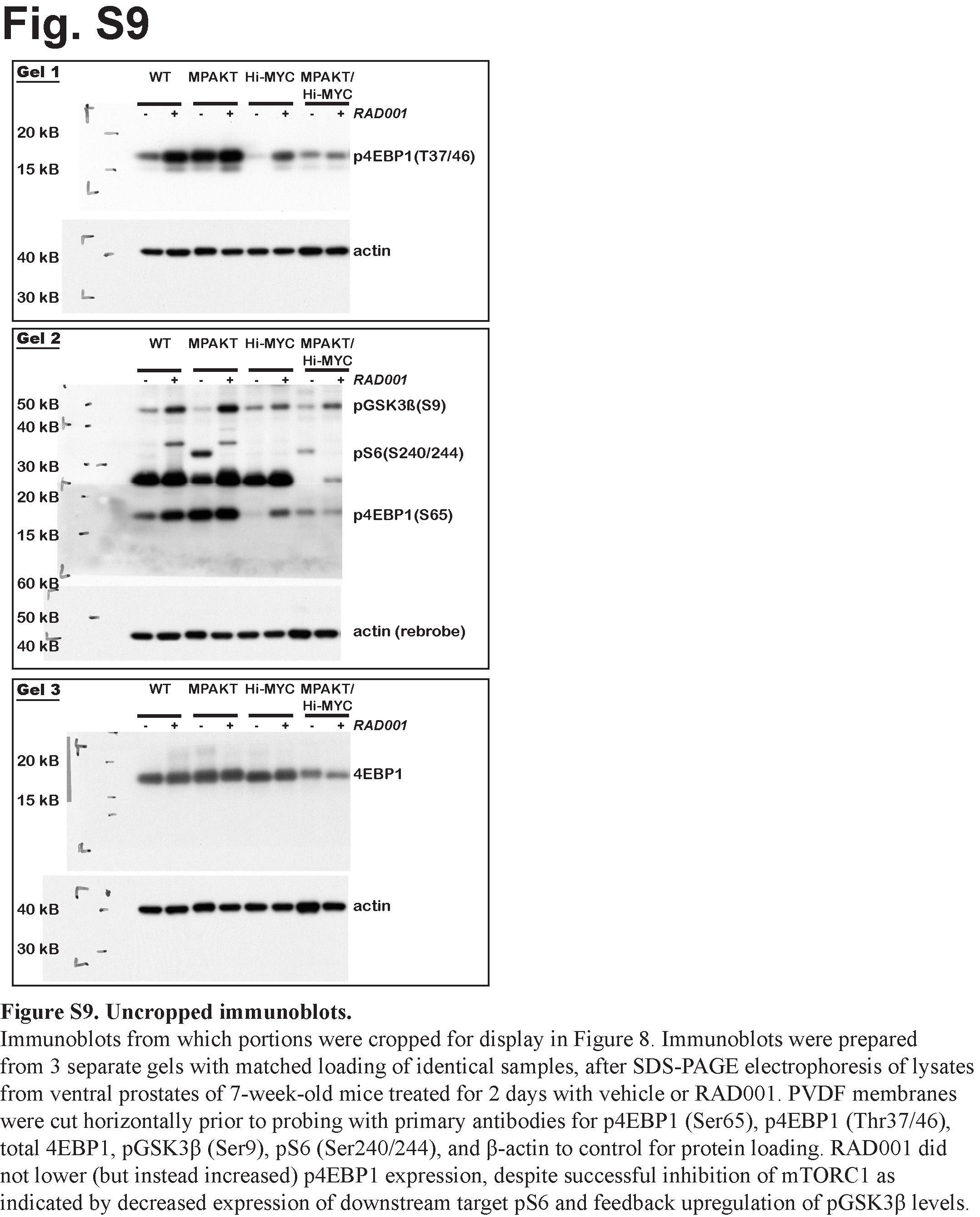

Supplement: Figure S9 — Uncropped immunoblots. Immunoblots from which portions were cropped for display in Figure 8. Immunoblots were prepared from 3 separate gels with matched loading of identical samples, after SDS-PAGE electrophoresis of lysates from ventral prostates of 7-week-old mice treated for 2 days with vehicle or RAD001. PVDF membranes were cut horizontally prior to probing with primary antibodies for p4EBP1 (Ser65), p4EBP1 (Thr37/46), total 4EBP1, pGSK3β (Ser9), pS6 (Ser240/244), and β-actin to control for protein loading. RAD001 did not lower (but instead increased) p4EBP1 expression, despite successful inhibition of mTORC1 as indicated by decreased expression of downstream target pS6 and feedback upregulation of pGSK3β levels. (TIF) [file pone.0017449.s009.tif]

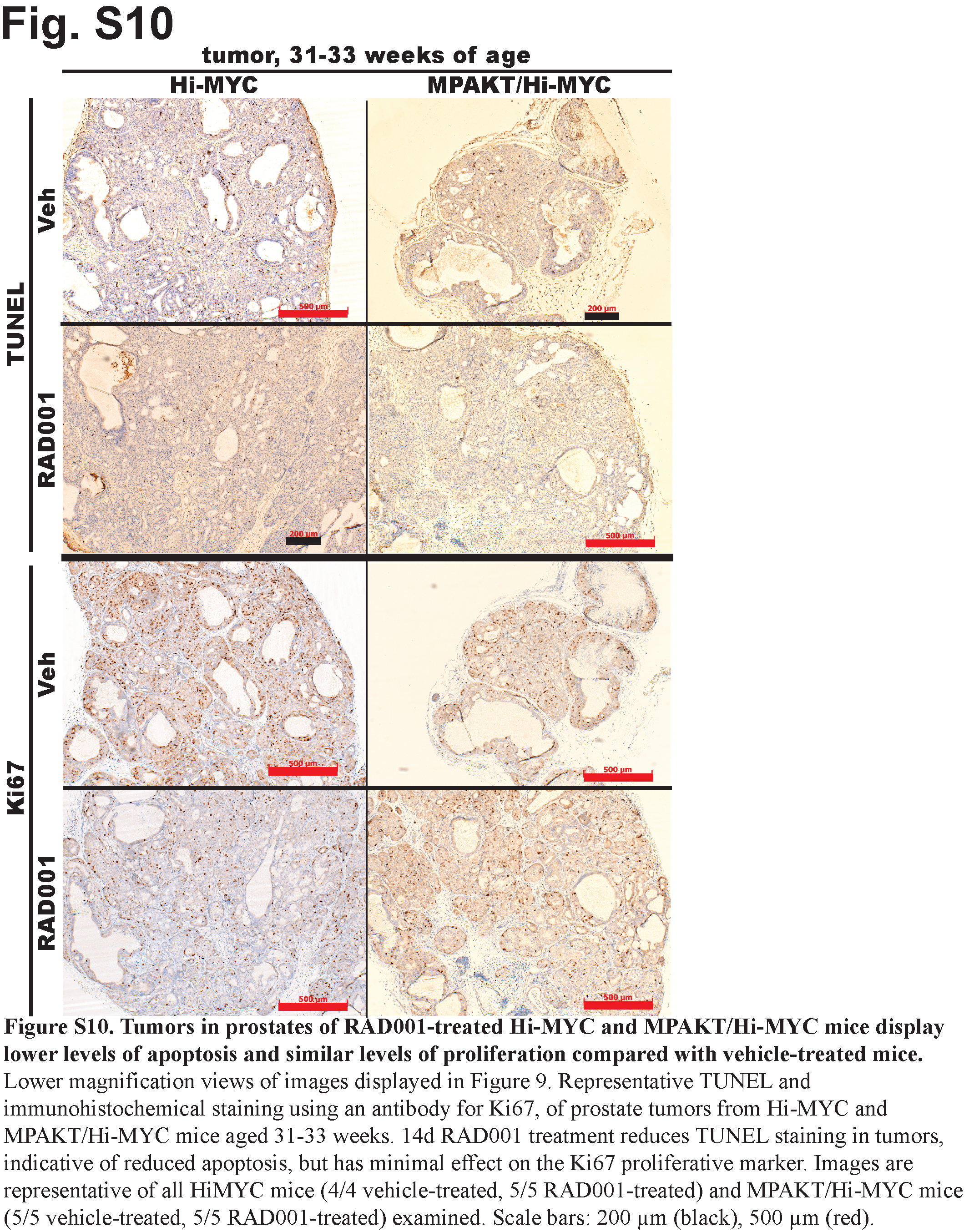

Supplement: Figure S10 — Tumors in prostates of RAD001-treated Hi-MYC and MPAKT/Hi-MYC mice display lower levels of apoptosis and similar levels of proliferation compared with vehicle-treated mice. Lower magnification views of images displayed in Figure 9. Representative TUNEL and immunohistochemical staining using an antibody for Ki67, of prostate tumors from Hi-MYC and MPAKT/Hi-MYC mice aged 31–33 weeks. 14d RAD001 treatment reduces TUNEL staining in tumors, indicative of reduced apoptosis, but has minimal effect on the Ki67 proliferative marker. Images are representative of all HiMYC mice (4/4 vehicle-treated, 5/5 RAD001-treated) and MPAKT/Hi-MYC mice (5/5 vehicle-treated, 5/5 RAD001-treated) examined. Scale bars: 200 µm (black), 500 µm (red). (TIF) [file pone.0017449.s010.tif]
