# Supplementary material for: MYC Cooperates with AKT in Prostate Tumorigenesis and Alters Sensitivity to mTOR Inhibitors
Source: PLoS One. 2011 Mar 4;6(3):e17449. doi: 10.1371/journal.pone.0017449 (PMC3048873; doi:10.1371/journal.pone.0017449)
Supplement: Table S1 — Primary copy number alternation data from array CGH analysis. (DOC) [file pone.0017449.s011.doc]

**Table S1**

**Primary copy number alternation data from array CGH analysis**

*Copy number*: 2 = multi copy gain, 1 = single copy gain, 0 = no alteration, -1 =heterozygous loss, -2 = homozygous loss

*Copy number alteration*: 1 = single- or multi-copy alteration in activating direction, 0 = no alteration in activating direction

|  |  | **Copy number** | | | | | **Copy number alteration** |
| --- | --- | --- | --- | --- | --- | --- | --- |
| **Case ID** |  | **MYC** | **PTEN** | **PIK3CA** | **AKT1** | **AKT3** | **PI3K pathway** |
| **PCA0001** | PRIMARY | 1 | 0 | 0 | 0 | 0 | 0 |
| **PCA0002** | PRIMARY | 1 | 0 | 0 | 0 | -1 | 0 |
| **PCA0003** | PRIMARY | 0 | 0 | 0 | 0 | 0 | 0 |
| **PCA0004** | PRIMARY | 0 | 0 | 0 | 0 | 0 | 0 |
| **PCA0005** | PRIMARY | 0 | 0 | 0 | 0 | 0 | 0 |
| **PCA0006** | PRIMARY | 0 | -1 | 0 | 0 | 0 | 1 |
| **PCA0007** | PRIMARY | -1 | 0 | 0 | 0 | 0 | 0 |
| **PCA0008** | PRIMARY | 0 | 0 | 0 | 0 | 0 | 0 |
| **PCA0009** | PRIMARY | 0 | 0 | 0 | 0 | 0 | 0 |
| **PCA0010** | PRIMARY | 1 | 0 | 0 | 0 | 0 | 0 |
| **PCA0011** | PRIMARY | 0 | 0 | 0 | 0 | 0 | 0 |
| **PCA0013** | PRIMARY | 0 | 0 | 0 | 0 | 0 | 0 |
| **PCA0014** | PRIMARY | 0 | 0 | -1 | 0 | 0 | 0 |
| **PCA0015** | PRIMARY | 0 | 0 | 0 | 0 | 0 | 0 |
| **PCA0016** | PRIMARY | 0 | 0 | 0 | 0 | 0 | 0 |
| **PCA0017** | PRIMARY | 0 | 0 | 0 | 0 | 0 | 0 |
| **PCA0018** | PRIMARY | 0 | 0 | 0 | 0 | 1 | 1 |
| **PCA0019** | PRIMARY | 0 | 0 | 0 | 0 | 0 | 0 |
| **PCA0020** | PRIMARY | 0 | 0 | 0 | 0 | 0 | 0 |
| **PCA0021** | PRIMARY | 0 | 0 | 1 | 0 | 0 | 1 |
| **PCA0023** | PRIMARY | 0 | 0 | 0 | 0 | 0 | 0 |
| **PCA0024** | PRIMARY | 0 | 0 | 0 | 0 | 0 | 0 |
| **PCA0025** | PRIMARY | 0 | 0 | 0 | 0 | 0 | 0 |
| **PCA0026** | PRIMARY | 0 | -1 | 0 | 0 | 0 | 1 |
| **PCA0027** | PRIMARY | 0 | 0 | 0 | 0 | 0 | 0 |
| **PCA0028** | PRIMARY | 0 | 0 | 0 | 0 | 0 | 0 |
| **PCA0029** | PRIMARY | 0 | 0 | 0 | 0 | 0 | 0 |
| **PCA0031** | PRIMARY | 0 | 0 | 0 | 0 | 0 | 0 |
| **PCA0032** | PRIMARY | 1 | 0 | 0 | 0 | 0 | 0 |
| **PCA0033** | PRIMARY | 0 | 0 | 0 | 0 | 0 | 0 |
| **PCA0034** | PRIMARY | 0 | 0 | 0 | 0 | 0 | 0 |
| **PCA0035** | PRIMARY | 0 | 0 | 0 | 0 | 0 | 0 |
| **PCA0036** | PRIMARY | 0 | 0 | 0 | 0 | 0 | 0 |
| **PCA0037** | PRIMARY | 0 | 0 | 0 | 0 | 0 | 0 |
| **PCA0038** | PRIMARY | 0 | 0 | 0 | 0 | 0 | 0 |
| **PCA0039** | PRIMARY | 0 | 0 | 0 | 0 | 0 | 0 |
| **PCA0040** | PRIMARY | 0 | 0 | 0 | 0 | 0 | 0 |
| **PCA0041** | PRIMARY | 0 | -1 | 0 | 0 | 0 | 1 |
| **PCA0042** | PRIMARY | 2 | 0 | 0 | 0 | 0 | 0 |
| **PCA0043** | PRIMARY | 0 | 0 | 0 | 0 | 0 | 0 |
| **PCA0044** | PRIMARY | 1 | 0 | 0 | 0 | 0 | 0 |
| **PCA0045** | PRIMARY | 0 | 0 | 0 | 0 | 0 | 0 |
| **PCA0046** | PRIMARY | 0 | 0 | 0 | 0 | 0 | 0 |
| **PCA0047** | PRIMARY | 0 | 0 | 0 | 0 | 0 | 0 |
| **PCA0048** | PRIMARY | 0 | 0 | 0 | 0 | 0 | 0 |
| **PCA0049** | PRIMARY | 0 | -2 | 0 | 0 | 0 | 1 |
| **PCA0050** | PRIMARY | 0 | 0 | 0 | 0 | 0 | 0 |
| **PCA0052** | PRIMARY | 1 | 0 | 0 | 0 | 0 | 0 |
| **PCA0053** | PRIMARY | -1 | 0 | 0 | 0 | 0 | 0 |
| **PCA0054** | PRIMARY | 0 | 0 | 0 | 0 | 0 | 0 |
| **PCA0055** | PRIMARY | 0 | 0 | 0 | 0 | 0 | 0 |
| **PCA0057** | PRIMARY | 0 | 0 | 0 | 0 | 0 | 0 |
| **PCA0058** | PRIMARY | 0 | 0 | 0 | 0 | 0 | 0 |
| **PCA0059** | PRIMARY | 0 | -1 | 0 | 0 | 0 | 1 |
| **PCA0060** | PRIMARY | 0 | -1 | 0 | 0 | 0 | 1 |
| **PCA0061** | PRIMARY | 0 | 0 | 0 | 0 | 0 | 0 |
| **PCA0062** | PRIMARY | 0 | 0 | 0 | 0 | 0 | 0 |
| **PCA0063** | PRIMARY | 1 | 0 | 0 | 1 | -1 | 1 |
| **PCA0064** | PRIMARY | 0 | 0 | 0 | 0 | 0 | 0 |
| **PCA0065** | PRIMARY | 0 | 0 | 0 | 0 | 0 | 0 |
| **PCA0066** | PRIMARY | 0 | 0 | 0 | 0 | 0 | 0 |
| **PCA0068** | PRIMARY | 0 | 0 | 0 | 0 | 0 | 0 |
| **PCA0069** | PRIMARY | 0 | 0 | 0 | 0 | 0 | 0 |
| **PCA0070** | PRIMARY | 0 | 0 | 0 | 0 | 0 | 0 |
| **PCA0071** | PRIMARY | 0 | 0 | 0 | 0 | 0 | 0 |
| **PCA0072** | PRIMARY | 0 | 0 | 0 | 0 | 0 | 0 |
| **PCA0073** | PRIMARY | 0 | -2 | 0 | 0 | 0 | 1 |
| **PCA0074** | PRIMARY | 0 | 0 | 0 | 0 | 0 | 0 |
| **PCA0075** | PRIMARY | 0 | 0 | 0 | 0 | 0 | 0 |
| **PCA0076** | PRIMARY | 0 | 0 | 0 | 0 | 0 | 0 |
| **PCA0078** | PRIMARY | 0 | 0 | 0 | 0 | 0 | 0 |
| **PCA0079** | PRIMARY | 0 | -2 | 0 | 0 | 0 | 1 |
| **PCA0080** | PRIMARY | 1 | 0 | 1 | 0 | 0 | 1 |
| **PCA0081** | PRIMARY | 0 | 0 | 0 | 0 | 0 | 0 |
| **PCA0082** | PRIMARY | 0 | 0 | 0 | 0 | 0 | 0 |
| **PCA0083** | PRIMARY | 0 | -2 | 0 | 0 | 0 | 1 |
| **PCA0084** | PRIMARY | 0 | 0 | 0 | 0 | 0 | 0 |
| **PCA0085** | PRIMARY | 0 | 0 | 0 | 0 | 0 | 0 |
| **PCA0086** | PRIMARY | 0 | 0 | 0 | 0 | 0 | 0 |
| **PCA0087** | PRIMARY | 1 | 0 | 0 | 0 | 0 | 0 |
| **PCA0088** | PRIMARY | 0 | 0 | 0 | 0 | 0 | 0 |
| **PCA0089** | PRIMARY | 0 | 0 | 0 | 0 | 0 | 0 |
| **PCA0090** | PRIMARY | 0 | 0 | 0 | 0 | 0 | 0 |
| **PCA0091** | PRIMARY | 0 | 0 | 0 | 0 | 0 | 0 |
| **PCA0092** | PRIMARY | 0 | -1 | 0 | 0 | 0 | 1 |
| **PCA0094** | PRIMARY | 1 | 0 | 0 | 0 | 0 | 0 |
| **PCA0095** | PRIMARY | 0 | 0 | 0 | 0 | 0 | 0 |
| **PCA0096** | PRIMARY | 0 | 0 | 0 | 0 | 0 | 0 |
| **PCA0097** | PRIMARY | 0 | 0 | 0 | 0 | 0 | 0 |
| **PCA0098** | PRIMARY | 0 | 0 | 0 | 0 | 0 | 0 |
| **PCA0099** | PRIMARY | 0 | 0 | 0 | 0 | 0 | 0 |
| **PCA0100** | PRIMARY | 0 | 0 | 0 | 0 | 0 | 0 |
| **PCA0102** | PRIMARY | 0 | 0 | -1 | 0 | 0 | 0 |
| **PCA0103** | PRIMARY | 0 | 0 | 0 | 0 | 0 | 0 |
| **PCA0104** | PRIMARY | 0 | -2 | 0 | 0 | 0 | 1 |
| **PCA0106** | PRIMARY | 0 | 0 | 0 | 0 | 0 | 0 |
| **PCA0108** | PRIMARY | 0 | 0 | 0 | 0 | 0 | 0 |
| **PCA0109** | PRIMARY | 0 | 0 | 0 | 0 | 0 | 0 |
| **PCA0110** | PRIMARY | 0 | 0 | 0 | 0 | 0 | 0 |
| **PCA0112** | PRIMARY | 0 | 0 | 0 | 0 | 0 | 0 |
| **PCA0114** | PRIMARY | 1 | -2 | 0 | 0 | 0 | 1 |
| **PCA0115** | PRIMARY | 0 | -1 | 0 | 0 | 0 | 1 |
| **PCA0116** | PRIMARY | 0 | 0 | 0 | 0 | 0 | 0 |
| **PCA0117** | PRIMARY | 0 | 0 | 0 | 0 | 0 | 0 |
| **PCA0118** | PRIMARY | 0 | 0 | 0 | 0 | 0 | 0 |
| **PCA0119** | PRIMARY | 1 | 0 | 0 | 0 | 0 | 0 |
| **PCA0121** | PRIMARY | 0 | 0 | 0 | 0 | 0 | 0 |
| **PCA0122** | PRIMARY | 0 | 0 | 0 | 0 | 0 | 0 |
| **PCA0123** | PRIMARY | 0 | 0 | 0 | 0 | 0 | 0 |
| **PCA0125** | PRIMARY | 0 | -1 | 0 | 0 | 0 | 1 |
| **PCA0126** | PRIMARY | 0 | -1 | 0 | 0 | 0 | 1 |
| **PCA0127** | PRIMARY | 0 | 0 | 0 | 0 | -1 | 0 |
| **PCA0128** | PRIMARY | 0 | -2 | 0 | 0 | 0 | 1 |
| **PCA0129** | PRIMARY | 0 | 0 | 0 | 0 | 0 | 0 |
| **PCA0130** | PRIMARY | 0 | 0 | 1 | 0 | 0 | 1 |
| **PCA0131** | PRIMARY | 0 | 0 | 0 | 0 | 0 | 0 |
| **PCA0132** | PRIMARY | 0 | 0 | 0 | 0 | 0 | 0 |
| **PCA0133** | PRIMARY | 0 | 0 | 0 | 0 | 0 | 0 |
| **PCA0135** | PRIMARY | 2 | 0 | 0 | 0 | 0 | 0 |
| **PCA0136** | PRIMARY | 1 | 0 | 0 | 0 | 0 | 0 |
| **PCA0137** | PRIMARY | 0 | 0 | 0 | 0 | 0 | 0 |
| **PCA0138** | PRIMARY | 0 | 0 | 0 | 0 | 0 | 0 |
| **PCA0139** | PRIMARY | 0 | -2 | 0 | 0 | 0 | 1 |
| **PCA0140** | PRIMARY | 1 | -1 | 0 | -1 | 1 | 1 |
| **PCA0141** | PRIMARY | 0 | 0 | 0 | 0 | 0 | 0 |
| **PCA0142** | PRIMARY | 0 | 0 | 0 | 0 | 0 | 0 |
| **PCA0143** | PRIMARY | 0 | 0 | 0 | 0 | 0 | 0 |
| **PCA0144** | PRIMARY | 0 | 0 | 0 | 0 | 0 | 0 |
| **PCA0145** | PRIMARY | 0 | 0 | 0 | 0 | 0 | 0 |
| **PCA0148** | PRIMARY | 0 | 0 | 0 | 0 | 0 | 0 |
| **PCA0149** | PRIMARY | 1 | 0 | -1 | 0 | 0 | 0 |
| **PCA0150** | PRIMARY | 0 | 0 | 0 | 0 | 0 | 0 |
| **PCA0151** | PRIMARY | 0 | 0 | 0 | 0 | 0 | 0 |
| **PCA0152** | PRIMARY | 0 | 0 | 0 | 0 | 0 | 0 |
| **PCA0153** | PRIMARY | 0 | 0 | 0 | 0 | 0 | 0 |
| **PCA0154** | PRIMARY | 0 | 0 | 0 | 0 | 0 | 0 |
| **PCA0155** | PRIMARY | 0 | 0 | 0 | 0 | 0 | 0 |
| **PCA0156** | PRIMARY | 2 | 0 | 0 | 0 | 0 | 0 |
| **PCA0157** | PRIMARY | 0 | 0 | 0 | 0 | 0 | 0 |
| **PCA0158** | PRIMARY | 0 | 0 | 0 | 0 | 0 | 0 |
| **PCA0160** | PRIMARY | 0 | 0 | 0 | 0 | 0 | 0 |
| **PCA0161** | PRIMARY | 0 | -1 | 0 | 0 | -1 | 1 |
| **PCA0162** | PRIMARY | 0 | 0 | 0 | 0 | 0 | 0 |
| **PCA0163** | PRIMARY | 0 | 0 | 0 | 0 | 0 | 0 |
| **PCA0164** | PRIMARY | 0 | 0 | 0 | 0 | 0 | 0 |
| **PCA0166** | PRIMARY | 0 | 0 | 0 | 0 | 0 | 0 |
| **PCA0167** | PRIMARY | 0 | 0 | 0 | 0 | 0 | 0 |
| **PCA0168** | PRIMARY | 0 | 0 | 0 | 0 | 0 | 0 |
| **PCA0171** | PRIMARY | 0 | -2 | 0 | 0 | 0 | 1 |
| **PCA0172** | PRIMARY | 0 | 0 | 0 | 0 | 0 | 0 |
| **PCA0173** | PRIMARY | 1 | 0 | 0 | 0 | 0 | 0 |
| **PCA0174** | PRIMARY | 1 | 0 | 0 | 0 | 0 | 0 |
| **PCA0177** | PRIMARY | 0 | 0 | 0 | 0 | 0 | 0 |
| **PCA0178** | PRIMARY | 0 | 0 | 0 | 0 | 0 | 0 |
| **PCA0179** | PRIMARY | 0 | 0 | 0 | 0 | 0 | 0 |
| **PCA0180** | PRIMARY | 0 | 0 | 0 | 0 | 0 | 0 |
| **PCA0181** | PRIMARY | 0 | -1 | 0 | 0 | 0 | 1 |
| **PCA0182** | MET | 0 | -2 | 0 | -1 | 0 | 1 |
| **PCA0183** | MET | -1 | -2 | 0 | -1 | 0 | 1 |
| **PCA0184** | MET | 0 | -2 | 1 | -1 | 0 | 1 |
| **PCA0185** | MET | 0 | -2 | 1 | -1 | 0 | 1 |
| **PCA0186** | MET | 2 | -2 | 1 | 1 | 0 | 1 |
| **PCA0187** | MET | 0 | 0 | 0 | 0 | 0 | 0 |
| **PCA0188** | MET | 2 | -1 | 0 | 0 | -1 | 1 |
| **PCA0189** | MET | 0 | 0 | 0 | 0 | 0 | 0 |
| **PCA0190** | MET | 1 | 0 | 0 | 1 | 1 | 1 |
| **PCA0191** | MET | 1 | -2 | -1 | -1 | 0 | 1 |
| **PCA0192** | MET | 0 | -1 | 0 | 0 | 0 | 1 |
| **PCA0193** | MET | 2 | -2 | 0 | 0 | 0 | 1 |
| **PCA0194** | MET | 1 | 0 | 0 | 0 | 0 | 0 |
| **PCA0195** | MET | 2 | -2 | -1 | 1 | 0 | 1 |
| **PCA0196** | MET | 2 | 0 | 1 | 0 | 0 | 1 |
| **PCA0197** | MET | 2 | 0 | 0 | 1 | -1 | 1 |
| **PCA0198** | MET | 0 | 0 | -1 | 0 | 0 | 0 |
| **PCA0199** | MET | 1 | -1 | 0 | 0 | 0 | 1 |
| **PCA0200** | MET | 0 | 0 | 0 | 0 | 0 | 0 |
| **PCA0201** | MET | 0 | 0 | 0 | 0 | 0 | 0 |
| **PCA0202** | MET | 1 | 0 | 0 | 0 | 0 | 0 |
| **PCA0203** | MET | 0 | 0 | 0 | 0 | 0 | 0 |
| **PCA0204** | MET | 2 | 1 | 0 | 0 | 2 | 1 |
| **PCA0205** | MET | 1 | -2 | 1 | -1 | -1 | 1 |
| **PCA0206** | MET | 0 | 0 | 0 | 0 | 0 | 0 |
| **PCA0207** | MET | -1 | -1 | 0 | -1 | 0 | 1 |
| **PCA0208** | MET | 1 | -2 | 0 | -1 | 1 | 1 |
| **PCA0209** | MET | 1 | -2 | 0 | 0 | 1 | 1 |
| **PCA0210** | MET | 0 | -2 | 1 | 1 | 0 | 1 |
| **PCA0211** | MET | 2 | 0 | 1 | -1 | 1 | 1 |
| **PCA0212** | MET | 1 | 0 | 0 | 0 | 0 | 0 |
| **PCA0213** | MET | 0 | 0 | 0 | 0 | 0 | 0 |
| **PCA0214** | MET | 0 | -2 | 0 | 0 | 1 | 1 |
| **PCA0215** | MET | 0 | -2 | 0 | 0 | 0 | 1 |
| **PCA0216** | MET | 1 | 1 | -1 | 0 | 1 | 1 |
| **PCA0217** | MET | 2 | -1 | 0 | 0 | 0 | 1 |
| **PCA0218** | MET | 0 | -2 | 1 | 0 | 0 | 1 |
