# Supplementary material for: MYC Cooperates with AKT in Prostate Tumorigenesis and Alters Sensitivity to mTOR Inhibitors
Source: PLoS One. 2011 Mar 4;6(3):e17449. doi: 10.1371/journal.pone.0017449 (PMC3048873; doi:10.1371/journal.pone.0017449)
Supplement: Text S1 — Supplemental Materials and Methods. (PDF) [file pone.0017449.s012.pdf]

__Text S1__

MYC cooperates with *AKT* in prostate tumorigenesis and alters sensitivity to mTOR inhibitors

Nicola J. Clegg, Suzana S. Couto, John Wongvipat, Haley Hieronymus, Brett S. Carver, Barry S. Taylor, Katharine Ellwood-Yen, William L. Gerald, Chris Sander, Charles L. Sawyers

Supplemental Materials and Methods

Comparative Genomic Hybridization Analysis of human tumors

The human prostate tissues analyzed in this study were from patients treated at Memorial Sloan-Kettering Cancer Center (MSKCC), all of whom provided informed written consent. The study was approved by the MSKCC Institutional Review Board and the MSKCC Human Tissue Utilization Committee. Copy number data from 194 high-quality primary and metastatic tumors was generated using the Agilent 244K aCGH array. aCGH arrays were processed and the resulting data was analyzed as previously described [1]. Briefly, primary and metastatic tumors were macro-dissected to ensure greater than 70 percent tumor content. The raw array data from tumor samples were processed by the RAE computational methodology (false discovery rate < 0.1) [2]. For all isoforms of autosomal genes in RefSeq (NCBI), copy number was discretized using the multi-component model in the RAE framework. Overlapping regions of the unified breakpoint profile, as inferred from segmentation, were used to assign one of five predicted aberration states to each isoform and tumor: (i) homozygous deletion ($D_1 \geq 0.9$), (ii) heterozygous loss ($D_0 \geq 0.9$ and $D_1 < 0.9$), (iii) copy-neutral (A_0 and $D_0 < 0.9$), (iv) single-copy gain ($A_0 \geq 0.9$ and $A_1 < 0.25$), and (v) multi-copy amplification ($A_0 \geq 0.9$ and

$A_1 \geq 0.25$). In the event of discontinuous coverage of a coding locus by regions that harbor intragenic breakpoints in copy-number segmentation, the region of extreme value in A_x and D_x, respectively, determined the assigned state. Using the aCGH data, tumors were assessed for genomic gain or amplification in *MYC*, *PIK3CA*, *AKT1*, *AKT2* and *AKT3*, and for losses in *PTEN* (Table S1). Copy number alterations were defined by aCGH as homozygous deletion, heterozygous loss, copy-neutral, single-copy gain or high level multi-copy amplification. *PIK3CA* amplification was defined as single- or multi-copy copy number amplification. PI3K pathway alteration was defined as *PTEN* deletion (hetero- or homozygous copy number deletion) and/or single- or multi-copy amplification of *AKT1*, *AKT2*, *AKT3* or *PIK3CA*. The significance of enrichment of these events was determined by Fisher's exact test (two-tailed). The complete aCGH dataset from these tumors is reported separately [3] and is available online at http://cbio.mskcc.org/prostate-portal/.

Generation and treatment of PTEN$^{pc-/-}$/Hi-MYC and MPAKT/Hi-MYC mice

Animal studies were carried out under protocol 06-07-012 approved by the MSKCC Institutional Animal Care and Use Committee. Institutional guidelines for the proper, humane use of animals in research were followed. Mice were housed in an animal facility maintained on a 12-hour light/dark cycle, at a constant temperature and relative humidity, with water and food available *ad libitum*. PTEN$^{pc-/-}$ mice (C57BL6/J background, PTEN$^{loxP/loxP}$/Pb-Cre4) were generated as previously described [4,5] using PTEN$^{loxP/loxP}$ [4] and Pb-Cre4 [6] mice. For the PTEN$^{pc-/-}$/Hi-MYC bigenic cross, Hi-MYC mice (FVB background, ARR2PB-Flag-*MYC*-PAI transgene) [7] previously generated in our laboratory were crossed into a C57BL6/J background and interbred, then crossed with

$PTEN^{loxP/loxP}$ mice. The $PTEN^{loxP/wt}$/Hi-Myc offspring (F1) were crossed to $PTEN^{loxP/loxP}$, generating $PTEN^{loxP/loxP}$/Hi-Myc offspring (F2), which in turn were crossed with $PTEN^{loxP/wt}$/Pb-Cre4 males generating the bigenic $PTEN^{pc-/-}$/Hi-MYC mice (F3) and littermates that were used for analysis. For generation of the bigenic MPAKT/Hi-MYC mice, MPAKT mice (FVB background, rPb-myr-HA-*AKT1* transgene) [8] were cross-bred with Hi-MYC mice. The F1 progeny were not used for subsequent breeding. Genotyping of pups was performed as previously described [4,5,7,8] by the MSKCC Mouse Genetics Core Colony Management service. All control mice used at each timepoint were from $PTEN^{pc-/-}$, MPAKT, Hi-MYC or wildtype littermates. Unless otherwise noted, mice in treatment cohorts were dosed for 2 weeks once daily by oral gavage at 10 mg/kg with either RAD001 emulsion (20 mg/mL) or placebo emulsion (Novartis Pharma AG).

Mouse dissections, tissue isolation, histology and pathology

Male mice were aged to the appropriate timepoint, treated if applicable, and then sacrificed for dissection. We evaluated prostate lesion progression (mPIN to adenocarcinoma) in a total of 114 mice including 30 MPAKT/Hi-MYC mice, and MPAKT, Hi-MYC and wild-type (WT) littermates. Prostates were harvested for analysis at ages 5-9 weeks, 16-20 weeks, and 6 months or greater. Urogenital organs were isolated and prostates were microdissected, separating the four pairs of prostate lobes (ventral, dorsal, lateral, and anterior) from one another and from the urethra. One lobe from each pair was flash frozen for later RNA isolation, while the remaining four lobes were oriented in an identical manner in 4-chamber cassettes, fixed in 10% phosphate-buffered formalin (Fisher SF100-4), processed and embedded in paraffin. The MSKCC Laboratory

of Comparative Pathology prepared 4 μm-thick serial tissue sections on charged glass slides. The tissues were stained with hematoxylin and eosin (H&E) for histologic analysis, or with terminal deoxynucleotidyl transferase dUTP nick end labeling (TUNEL) [9] for analysis of apoptosis, or were used for immunohistochemistry (IHC) for smooth muscle actin (Dako #M0851, 1:15000), E-cadherin (BD Transduction Laboratories #610182, 1:1000), mac-2 (Cedarlane #CL8942B, 1:100), B-220 (BD Pharmingen #550286, 1:200), CD3 (Dako #A045, 1:1000 or Vector Labs #VP-RM01, 1:500), phospho-AKT (Ser473) (Cell Signaling #3787, 1:25) and Ki67 (Vector Labs #VP-K451, 1:10000). Antigen retrieval was performed prior to immunohistochemical staining by microwave irradiation (high power, 15 minutes) in sodium citrate buffer (10 mM, pH 6.0), except in the case of B-220 staining for which no pre-treatment was necessary. The Vectastain ABC Elite Kit (Vector Labs # PK-6100) was used for secondary visualization of antigens following labeling with the primary antibody or IgG control. The MSKCC Molecular Cytology Core performed additional IHC using the Discovery XT processor (automated processing includes antigen retrieval with the CC1 Tris-EDTA-based reagent) and DABMap™ chromogenic detection kit (Ventana Medical Systems) for detection of ribosomal protein phospho-S6 (Ser235/236) (Cell Signaling #2211L, 0.03 μg/mL), phospho-AKT (Ser473) (Cell Signaling #4060, 1 μg/L), p27(Kip1) (BD Transduction Laboratories #610241, 0.5 μg/mL), MYC (N-term) (Epitomics #1472-1, 2μg/mL) [10], smooth muscle actin (Sigma #A5228, 1 μg/mL), collagen IV (AbD Serotec #2150-1470, 1 μg/mL), and phospho-4E-BP1 (Thr37/46) (236B4) (Cell signaling #2855, 0.05 μg/mL). The Vectastain Elite ABC Kit (Rabbit IgG) (Vector Labs #PK6101) was used for secondary visualization of antigens, except in the case of p27(Kip1) and smooth muscle

actin for which the Vector M.O.M. Basic Kit was used (Vector Labs #BMK-2202). Appropriate positive, negative and IgG controls were included in all IHC assays.

Microscopy and Imaging

Microscopy was performed using an Olympus BX45 microscope and images were captured using an Olympus DP12 or DP25 camera on a U-TVO.5XC attachment with Olympus DP2-BSW (version 2.2) software. Slides were scanned using the Mirax Scan 150 (Zeiss) with 20x/0.8NA objective and automatic exposure settings, and image files are available online at http://cbio.mskcc.org/Public/Sawyers_Clegg_AktMyc_2010/.

Quantitative Real-time RT-PCR

Tissues frozen for total RNA isolation were homogenized with a tissue grinder (in a tube containing one lobe each of the anterior, ventral, dorsal and lateral prostates from an individual mouse) in TRIzol Reagent (Invitrogen #15596-026), followed by phase separation, washing, precipitation and resuspension of RNA in water, all according to manufacturer's protocols. The RNA was further purified using the RNeasy kit (Qiagen #74106) and treated with on-column DNase (RNase-Free DNase Set, Qiagen #79254) according to manufacturer's protocols, followed by quantification and normalization using A_{260}/A_{280}. cDNA synthesis from 1 μg RNA was carried out using the TaqMan Reverse Transcription Reagents (Applied Biosystems #N808-0234) with random hexamers, according to the manufacturer's protocol. Triplicate samples for quantitative PCR were run in the Realplex MasterCycler (Eppendorf) using the Power SYBR Green PCR Mastermix (Applied Biosystems). Each reaction contained 1 μL of cDNA in a total volume of 20 μL. ΔC_t for each gene was determined after normalization to β-actin and $\Delta\Delta C_t$ was calculated relative to the designated reference sample. Gene expression values

were then expressed as a fold change, calculated by $2^{-\Delta\Delta Ct}$. Mouse *AKT1* and mouse *GAPDH* primers were obtained from SA Biosciences and used at 200 nM. Mouse *B-actin* and human *MYC* primers were used as previously described [11]. Primers for human *AKT1*, used at 600 nM final concentration, were designed with Primer3 [12]: forward - CTTCTTTGCCGGTATCGTGT and reverse - CTGGCCGAGTAGGAGAACTG.

Immunoblotting

Protein lysates were prepared by homogenization of frozen ventral prostates in T-PER protein extraction reagent (Pierce), using the FastPrep-24 system with Lysing Matrix A (MP Biomedicals). Protein concentrations were determined with the BCA Protein Assay Kit (Pierce). SDS-PAGE gel electrophoresis and transfer to an Immobilon PVDF-P membrane (Invitrogen) was performed with Novex Bis-Tris gels using the NuPage System (Invitrogen) according to manufacturer's protocols. Immunoblotting was performed overnight with primary antibody, followed after by a 45 minute incubation at room temperature with secondary antibody. Tris-buffered saline-Tween20 (TBS-T) was used for all washes. Chemiluminescent detection was performed with ECL or ECL-plus reagents (Amersham, GE Healthcare). Antibodies were diluted in a 5% bovine serum albumin solution in TBS-T; Cell signaling: 4E-BP1 (53H11) (#9644, 1:1000), phospho-4E-BP1 (Thr37/46) (236B4) (#2855, 1:1000), phospho-4E-BP1 (Thr70) (#9455, 1:1000), phospho-S6 ribosomal protein (Ser240/244) (#2215, 1:10,000), phospho-GSK-3β (Ser9) (5B3) (# 9323, 1:1000); β-Actin (AC-15) (Sigma #A1978, 1:20,000); peroxidase-conjugated AffiniPure secondary antibodies were from Jackson ImmunoResearch Laboratories.

References

1. (2008) Comprehensive genomic characterization defines human glioblastoma genes and core pathways. Nature 455: 1061-1068.
2. Taylor BS, Barretina J, Socci ND, Decarolis P, Ladanyi M, et al. (2008) Functional copy-number alterations in cancer. PLoS One 3: e3179.
3. Taylor BS, Schultz N, Hieronymus H, Gopalan A, Xiao Y, et al. (2010) Integrative genomic profiling of human prostate cancer. Cancer Cell 18: 11-22.
4. Trotman LC, Niki M, Dotan ZA, Koutcher JA, Di Cristofano A, et al. (2003) Pten dose dictates cancer progression in the prostate. Plos Biology 1: 385-396.
5. Chen Z, Trotman LC, Shaffer D, Lin HK, Dotan ZA, et al. (2005) Crucial role of p53-dependent cellular senescence in suppression of Pten-deficient tumorigenesis. Nature 436: 725-730.
6. Wu X, Wu J, Huang J, Powell WC, Zhang J, et al. (2001) Generation of a prostate epithelial cell-specific Cre transgenic mouse model for tissue-specific gene ablation. Mech Dev 101: 61-69.
7. Ellwood-Yen K, Graeber TG, Wongvipat J, Iruela-Arispe ML, Zhang J, et al. (2003) Myc-driven murine prostate cancer shares molecular features with human prostate tumors. Cancer Cell 4: 223-238.
8. Majumder PK, Yeh JJ, George DJ, Febbo PG, Kum J, et al. (2003) Prostate intraepithelial neoplasia induced by prostate restricted Akt activation: the MPAKT model. Proc Natl Acad Sci U S A 100: 7841-7846.

9. Gavrieli Y, Sherman Y, Ben-Sasson SA (1992) Identification of programmed cell death in situ via specific labeling of nuclear DNA fragmentation. J Cell Biol 119: 493-501.

10. Gurel B, Iwata T, Koh CM, Jenkins RB, Lan F, et al. (2008) Nuclear MYC protein overexpression is an early alteration in human prostate carcinogenesis. Mod Pathol 21: 1156-1167.

11. Watson PA, Ellwood-Yen K, King JC, Wongvipat J, Lebeau MM, et al. (2005) Context-dependent hormone-refractory progression revealed through characterization of a novel murine prostate cancer cell line. Cancer Res 65: 11565-11571.

12. Rozen S, Skaletsky H (2000) Primer3 on the WWW for general users and for biologist programmers. Methods Mol Biol 132: 365-386.
